# A point cloud segmentation framework for image-based spatial transcriptomics

**DOI:** 10.1038/s42003-024-06480-3

**Published:** 2024-07-06

**Authors:** Thomas Defard, Hugo Laporte, Mallick Ayan, Juliette Soulier, Sandra Curras-Alonso, Christian Weber, Florian Massip, José-Arturo Londoño-Vallejo, Charles Fouillade, Florian Mueller, Thomas Walter

**Affiliations:** 1 Centre for Computational Biology (CBIO), Mines Paris, PSL University, 75006 Paris, France; 2 Institut Curie, PSL University, 75005 Paris, France; 3 INSERM, U900, 75005 Paris, France; 4 Institut Pasteur, Université Paris Cité, Imaging and Modeling Unit, F-75015 Paris, France; 5 Institut Pasteur, Université Paris Cité, Photonic Bio-Imaging, Centre de Ressources et Recherches Technologiques (UTechS-PBI, C2RT), F-75015 Paris, France; 6 Institut Curie, Inserm U1021-CNRS UMR 3347, University Paris-Saclay, PSL Research University, Centre Universitaire, Orsay, Cedex France; 7 Institute of Cell Biology (Cancer Research), University Hospital Essen, Essen, Germany

**Keywords:** Image processing, Statistical methods

## Abstract

Recent progress in image-based spatial RNA profiling enables to spatially resolve tens to hundreds of distinct RNA species with high spatial resolution. It presents new avenues for comprehending tissue organization. In this context, the ability to assign detected RNA transcripts to individual cells is crucial for downstream analyses, such as in-situ cell type calling. Yet, accurate cell segmentation can be challenging in tissue data, in particular in the absence of a high-quality membrane marker. To address this issue, we introduce ComSeg, a segmentation algorithm that operates directly on single RNA positions and that does not come with implicit or explicit priors on cell shape. ComSeg is applicable in complex tissues with arbitrary cell shapes. Through comprehensive evaluations on simulated and experimental datasets, we show that ComSeg outperforms existing state-of-the-art methods for in-situ single-cell RNA profiling and in-situ cell type calling. ComSeg is available as a documented and open source pip package at https://github.com/fish-quant/ComSeg.

## Abstract (web summary)

ComSeg is a cell segmentation algorithm for image-based spatial transcriptomics. ComSeg operates directly on single RNA positions, leveraging nuclei positions, and is applicable to complex tissues with arbitrary cell shapes.

## Introduction

Understanding the spatial organization of tissues at the single-cell level is crucial to study disease and development^1–3^. Molecular profiling of single cells in their spatial context allows us to infer cell states and cell types, cell–cell interactions, and cell-fate decision-making^4^, as well as the study of the overall tissue architecture, under normal and diseased conditions, leading to the definition of spatial domains and disease signatures^5^.

Spatial transcriptomics denotes a large panel of technologies that allow to measure gene expression and to retrieve the spatial positions of the mRNA molecules. These methods can largely be divided into two groups: sequencing-based^6,7^ and imaging-based^8–10^

spatial transcriptomics (SST and IST, respectively). The former measures the entire transcriptome, but has a lower spatial resolution, while the latter relies on measuring a subset of genes at high resolution. Such a subset consists of pre-defined marker genes that are specific for the cell states or types of interest. Since IST captures only a subset of the transcriptome, we will refer to these approaches as RNA profiling. These imaging-based approaches are variants of single-molecule fluorescence in situ hybridization (smFISH) methodologies and can detect RNAs with single-molecule sensitivity with high spatial resolution, several orders of magnitude below the scale of a single cell. A typical dataset consists of 2D or 3D point clouds of the imaged RNA species. One challenge in the analysis of these data is the correct assignment of each RNA to its cell of origin. Indeed, in contrast to single-cell RNA sequencing (scRNA-seq), the information on which RNA molecules belong to the same cell has to be inferred from the image. This assignment is crucial, as it impacts cell type identification and, thus, the major aspect of the analysis we would like to perform.

One frequently used approach to perform this assignment is to segment the cells from additional channels employing markers for cell and nucleus segmentation. RNAs are then assigned to the cells based on their spatial positions with respect to this segmentation. Such stainings typically encompass labeling of the nucleus with DAPI, cellular staining with one or more cell membrane dyes, or labeling all RNAs as a proxy for the cytoplasm^10–12^. However, cell membrane staining is often not an option^13,14^. Besides, staining can be technically complex^15^, and may not work equally well for the entire tissue, thus leading to inhomogeneous cell segmentation and cell type calling accuracy. Further, the boundaries of individual cells can be challenging to segment, especially for tissue with complex 3D cellular morphologies.

More recently, several computational approaches have been presented to detect individual cells in the images and establish their RNA profile without relying on a dedicated cell marker. These methods rely only on the RNA positions and in some cases a DAPI stain, to regroup RNAs according to local transcription profiles. Such RNA point clouds can then act as an approximation for cell shape, by establishing a hull that encompasses all RNAs that were deemed to belong together^15–17^. Methods like pciSeq^16^ and Baysor^15^ leverage statistical models to group RNAs. pciSeq segments cells to match external scRNA-seq datasets and Baysor divides RNAs into spatially homogeneous areas. Recently, deep-learning approaches were proposed. JSTA^17^ and BIDCell^18^ train a cell membrane segmentation model exploiting nuclei segmentation and external scRNA-seq while SCS trains a membrane segmentation transformer network using solely nuclei segmentation as input. While these approaches hold great promise for the analysis of spatial RNA profiling data, their use is limited by implicit priors on cell shape^15,16^ leading to low performance in complex tissues, requirement of additional scRNA-seq data^16,17^ or manual annotations which are scarce and error-prone^18,19^ (see Table 1).

**Table 1:** Characteristics of existing methods for spatial RNA profiling

	Watershed	pciSeq^16^	JSTA^17^	SSAM^32^	Baysor^15^	BIDCell^18^	SCS^19^	ComSeg
Convex shape prior	Yes	Yes	Yes	No	Yes	Yes	No	No
Require external scRNA-seq dataset	No	Yes	Yes	No	No	Yes	No	No
Require nucleus positions	Yes	Yes	Yes	No	No	Yes	Yes	Yes
Single-cell profiling	Yes	Yes	Yes	No	Yes	Yes	Yes	Yes

While there already exist methods for spatial RNA profiling, these methods usually come with requirements that are not always met in practice. For instance, some methods require a high RNA density, which often implies a large panel of marker genes. However, while current commercial spatial RNA profiling approaches provide hundreds of marker genes, they remain extremely costly, and for many questions, fewer marker genes will be sufficient, which can be probed with simpler custom-built solutions^20,21^. Furthermore, most methods^15,16^ implicitly assume convex or even round cell shapes. In contrast, the cell shapes in some tissues can be complex and often deviate from such simple shapes. Approaches relying on strong assumptions on cell shape are, therefore, suboptimal for many of these tissue types. Lastly, some methods require parallel scRNA-seq data^16–18^, which in practice is not always readily available, so ideally the use of such information should be only optional.

To alleviate these issues, we propose a method named ComSeg. ComSeg uses as input only the coordinates of the RNA molecules and the nucleus positions obtained with staining such as DAPI. ComSeg then groups RNAs with similar expression profiles aided by these landmarks. It does not require scRNA-seq data or cytoplasmic markers, nor does it make implicit use of any prior assumptions regarding cell morphology. Instead, the method describes RNA point clouds as graphs weighted by gene co-expression and relies on graph community detection. Our method is easy to apply by design as it does not require complex machine learning model training. Furthermore, we provide the tool as an open-source Python package (https://github.com/fish-quant/ComSeg) with extensive documentation: https://comseg.readthedocs.io, compatible with the scverse environment^22^. To facilitate the application of ComSeg on large datasets, we ensured its compatibility with SOPA^23^, as documented on https://comseg.readthedocs.io.

Development of such analysis approaches requires annotated ground truth to assess their performance. Experimental ground truth can be obtained in some cases by employing membrane markers, from which the cytoplasmic membrane might be segmented with deep neural networks or manual annotation^24^. However, the ground truth quality is affected by the heterogeneity of the staining quality which may be low on complex and dense tissue^15^. Furthermore, experimental data does not permit a more systematic exploration of parameters such as the expression level or cell morphology. Hence, similarly to previous studies^16,17^, we address the lack of high-quality ground truth by generating simulated data, allowing us to control the complexity of the input data. We developed *SimTissue* (https://github.com/tdefa/SimTissue), an open-source Python simulation package to reproduce in silico fluorescent-based spatial transcriptomic experiments.

We used the *SimTissue* framework to validate ComSeg on simulated data with increasing complexity in terms of RNA abundance, the number of marker genes, and tissue morphology. ComSeg outperforms other methods in terms of the Jaccard index for RNA-cell association in most of the tested scenarios. We also validated ComSeg on an in-house experimental dataset of mouse lung tissue imaged with smFISH and human embryonic lung tissue^25^ imaged with HyBISS^26^. On these experimental data, ComSeg estimates accurate RNA profiles that match established scRNA-seq datasets. Finally, we benchmark ComSeg on two MERFISH^8^ datasets with cell membrane staining, the mouse ileum dataset^15^ and a human breast cancer dataset from VizGen from the MERSCOPE FFPE Human Immuno-Oncology Data Release. We leverage areas with high-quality membrane staining to obtain segmentation ground truth. Overall, the shape-agnostic approach of ComSeg demonstrates notable efficacy for complex tissue composed of cells with non-convex shapes.

## Results

### ComSeg overview

**Fig. 1:**
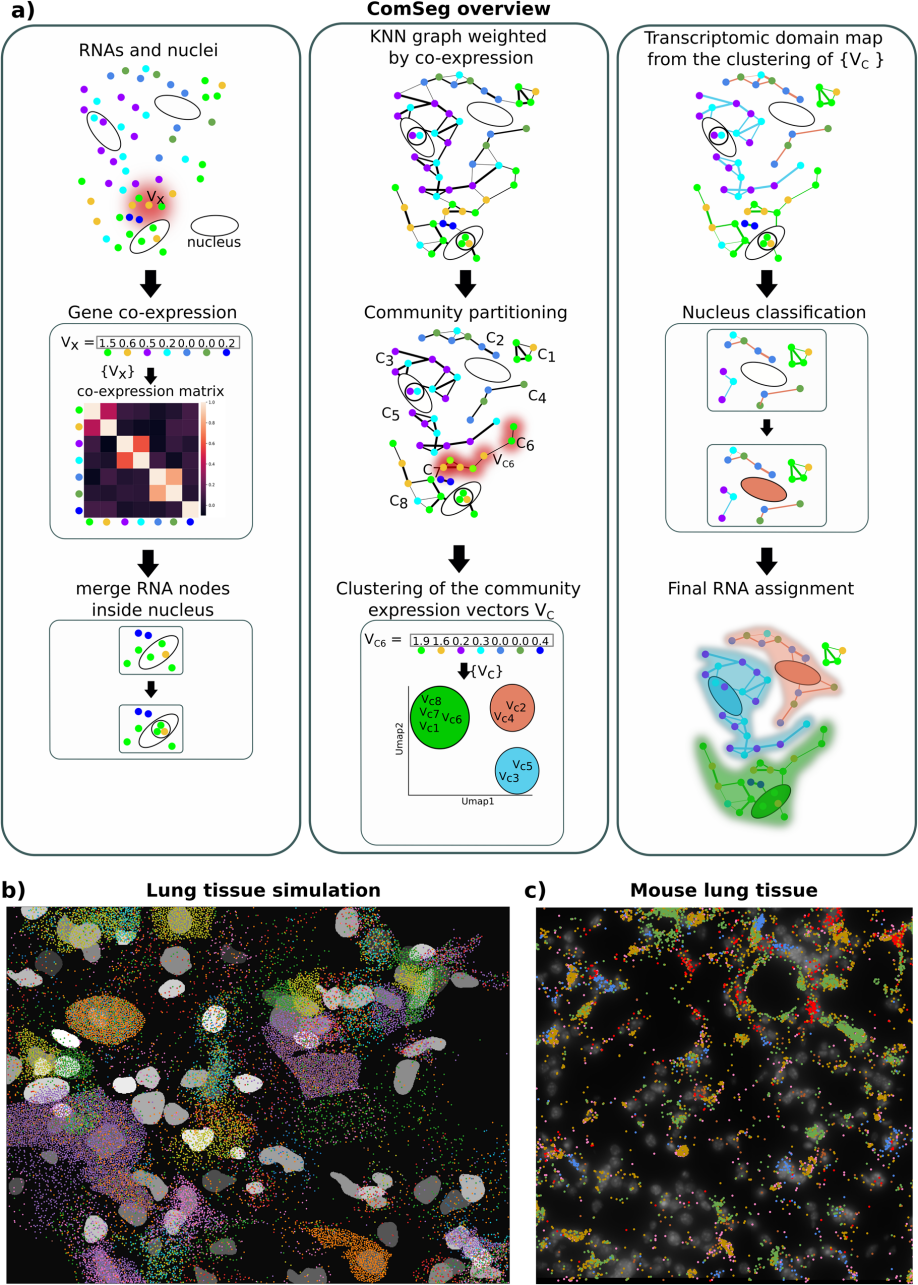
Overview of the method and simulated and experimental data. **a** Overview of the ComSeg method. **b** 3D lung tissue simulation with 34 marker genes represented by different colors. **c** Mouse Lung tissue from our in-house dataset with six marker genes represented by different colors.

Here we present ComSeg, a graph-based method to perform cell segmentation from spatial RNA profiling data. The method operates directly on RNA point clouds and leverages nuclear staining to increase segmentation quality. For this, we define a KNN graph, where each RNA molecule is a node and where edges are weighted according to the co-expression score of the corresponding genes. Instead of relying on external data to compute these co-expression weights, we leverage the input images by estimating the local gene expression vector in a close environment of each RNA molecule (see Material and Methods). We then detect groups of RNA molecules with similar gene expression in their local environment, by the use of a modified version of the Louvain community detection method^27^. Our community detection method can leverage spatial landmarks like DAPI segmentation as prior knowledge. Lastly, we group the detected RNA communities with similar expression profiles using the Leiden algorithm^28^ and obtain a transcriptomic domain map at the tissue scale. To obtain single-cell RNA profiles, we exploit the nucleus position concurrently with the transcriptomic domain map. An overview of the method is displayed in Fig. 1a.

### State-of-the-art methods for cell segmentation

We benchmarked ComSeg against methods that can be used in an equivalent setting i.e., single-cell spatial RNA profiling approaches requiring no external dataset. As a first baseline, we calculated the Watershed transformation^29^ as this method is often used for RNA-nuclei association in tissue^30,31^. This method effectively calculates a Voronoi tessellation with the nuclear regions as markers with an additional distance constraint, and is thus equivalent to assigning RNA transcripts to their nearest nucleus. The method, hence, perfectly works for convex cells of homogeneous size if all nuclei are detected, but may fail otherwise. Second, we benchmarked Baysor^32^, a cell marker-free segmentation method optimizing the joint likelihood of transcriptional composition and prior cell morphology. It is particularly suited for cases where only nuclear staining or weak cytoplasmic staining are available. Baysor uses an elliptic function as cell shape prior. We also applied a method leveraging external scRNA-seq data, pciSeq^16^, a Bayesian model leveraging prior scRNA-Seq data to estimate a probability of cell assignment for each read. Given the observed RNA spot configuration, the method performs cell assignment to match known transcriptomic profiles from scRNA-seq. Hence, it also simultaneously assigns each cell to a cell type. Leveraging prior knowledge may help pciSeq avoid wrong RNA-cell assignment. However, pciSeq implicitly uses a spherical cell shape prior that may hinder its application on complex tissue. Finally, we benchmarked our method against SCS^19^, a recently published deep-learning-based method primarily designed for Stereo-seq data, but also applicable to IST data. The method trains transformers to predict, for each transcriptomic spot measurement, the direction to the nucleus it belongs to.

All of these methods require hyperparameter tuning. For the Watershed method, we had to set the maximum distance parameter to avoid the incorrect assignment of RNA to nuclei that were too distant. This maximum distance parameter had to be optimized to account for differences in tissue complexity and cell density. For pciSeq and Baysor, we kept the default settings for all experiments, with the exception of the scaling parameter for Baysor, which was automatically set using prior nucleus segmentation. For the analysis of the mouse ileum with Baysor, we used the compartment-specific gene list provided^15^ as additional parameters. SCS was trained over 100 epochs with a bin size of 15 pixels for all the experiments. Lastly, the mean cell size parameter of ComSeg was manually adjusted for each dataset. More details regarding hyperparameter settings of the benchmarked methods can be found in Supplementary Note 1.

### Benchmark on simulation of lung tissue

To benchmark the methods on challenging data, we first turned to simulations mimicking lung tissue (see Methods). Here, cells have complex shapes in 3D, and airways add empty space devoid of any transcripts. Moreover, we also simulated some cells without nuclei, which can occur in tissue sections. Further, we sampled real expression profiles from our recent scRNA-seq data^33^. We simulated 34 cell-type marker genes selected with the NS-forest algorithm^34^. This marker list enabled us to classify 19 different cell types present in lung tissues with an accuracy of 0.88 for cell type calling when having a perfect RNA assignment to cells (Methods).

To quantitatively compare the different methods, we implemented different metrics (see Methods). We assessed the quality of the RNA-cell assignment with the mean Jaccard index per cell, which is calculated for the RNA positions.

The ultimate goal of all the methods is to obtain a gene expression vector for each cell. In this context, not all types of RNA-cell association errors are equivalent. If a cell misses some RNAs, it may still be possible to accurately assess its RNA profile, similar to cell type classification in scRNA-seq, where only a fraction of all RNAs are sequenced. However, if some RNAs are wrongly associated with a cell, this can create a mixed expression profile resulting in incorrect cell type classification. We thus reported the mean percentage of wrongly associated RNA per cell (WA) and the mean percentage of missing RNA per cell (MS) separately. Lastly, we assessed cell type calling accuracy by comparing each method’s results to the ground truth cell type defined by scRNA-seq data. To classify cell types, we computed the cosine distance between predicted cell expression vectors derived from images and the scRNA-seq cell type cluster centroid (see Methods).

**Fig. 2:**
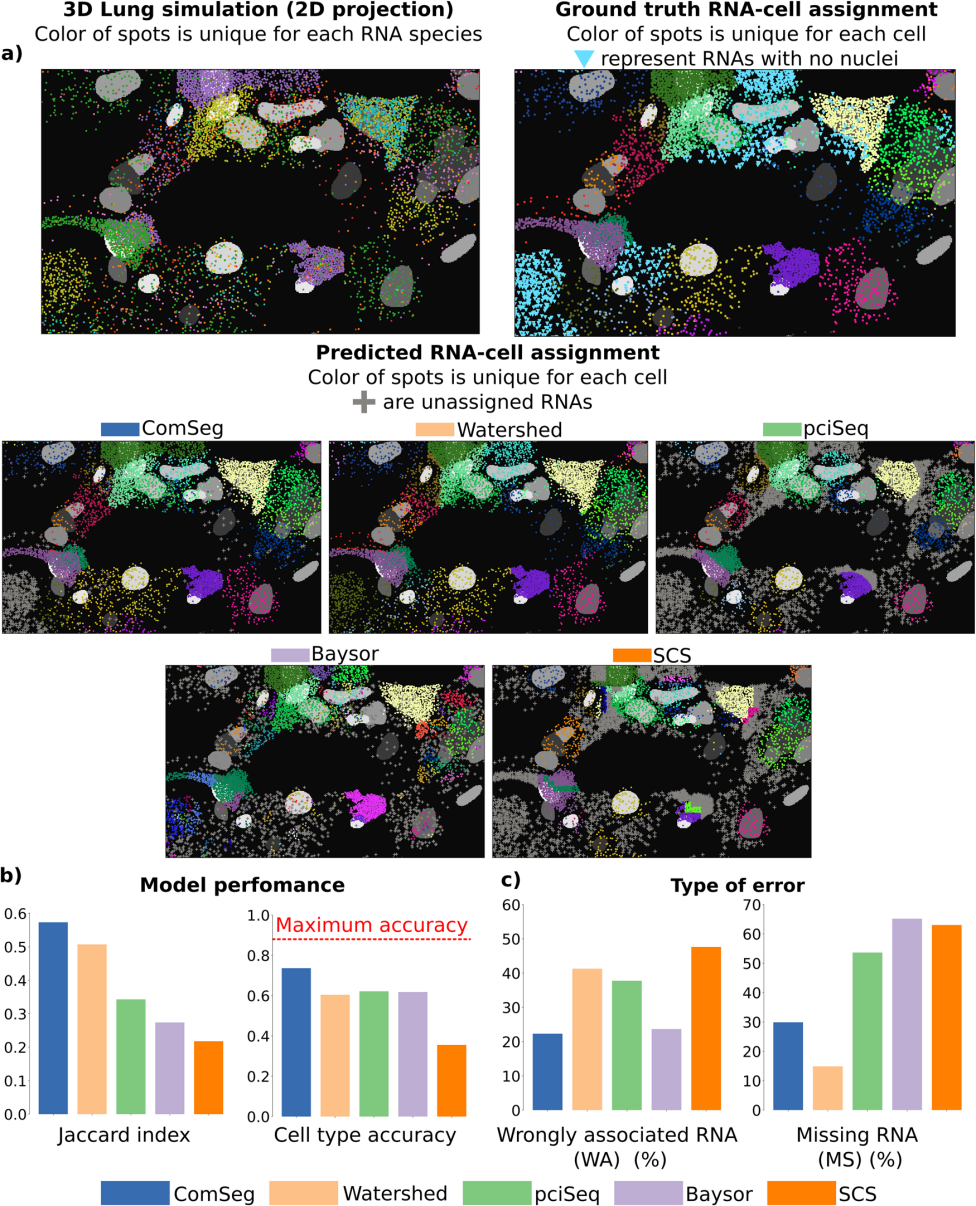
Benchmarking on simulated lung tissue. **a** 3D lung simulation and RNA assignment of the different models. The ground truth takes into account only cells with a nucleus. **b** Performance metrics of the benchmarked models with the mean Jaccard index per cell and cell type calling accuracy, the red line is the maximum accuracy when having a perfect RNA assignment to cells. **c** Error metrics of the benchmarked models with the mean percentage of wrongly associated RNA per cell (*WA*) and mean percentage of missing RNA per cell (*MS*).

Examples of RNA-cell assignment from the different approaches on lung tissue simulation are shown in Fig. 2a. ComSeg outperforms Baysor, pciSeq, SCS, and the Watershed algorithm with a Jaccard index of 0.57 against 0.30, 0.33, 0.21, and 0.50 for Baysor, pciSeq, SCS, and Watershed, respectively (Fig. 2b, left panel). The lower Jaccard index of SCS could be attributed to its original design for sequencing-based data with access to the complete transcriptome, whereas in our case, we only simulate a limited number of markers. Furthermore, SCS does not utilize the 3D information provided by the simulation and the spatial resolution must be reduced to accommodate SCS to image data. A closer look at the results reveals that the type of error is not the same for the four models. Baysor, SCS and pciSeq have a high percentage (more than 50%) of missing RNA per cell (Fig. 2c, right panel) while Watershed has a very low (15%) mean percentage of missing RNA per cell. This is not surprising, as the Watershed computes a Voronoi tessellation and hence assigns all RNAs except those that are very far from nuclei. Watershed, SCS, and pciSeq have a higher mean percentage of wrongly associated RNA per cell (about 40%), while it is low for Baysor and ComSeg (roughly 20% on average) (Fig. 2c, left panel).

Next, we compared the estimated expression profiles from the simulated images to the known, underlying scRNA-seq ground truth, by performing cell type calling. Here, ComSeg reaches 74% accuracy, compared to 60, 62, 35, and 62% for Watershed, pciSeq, SCS, and Baysor respectively (Fig. 2b, right panel). The higher accuracy of ComSeg for cell-type calling can be explained by the lower misassociation error rate (Fig. 2c, left panel). Of note, the best cell type calling accuracy that could be achieved is 88%, which is thus the value that would be reached if all RNAs were correctly assigned to the cell they belong to. This value is not 100% because the selected marker genes do not perfectly recapitulate the full transcriptomic profiles. On the other hand, we observe that cell-type calling is heavily impacted by wrong RNA assignments.

In conclusion, ComSeg performs better in terms of RNA-cell association and substantially improves downstream tasks like cell type calling on lung tissue simulation compared to the current state-of-the-art. In view of these results, we next turned in Supplementary Note 2 to simulations with a simpler tissue geometry, in order to better understand the limitations of each method.

### Application to experimental data without cell membrane staining

We applied ComSeg to two different experimental lung datasets with solely nuclei staining, and each with a specific challenge for the analysis. The first dataset was created in-house. In this experiment, we visualized 6 different marker genes in 3D mouse lung tissue. Our approach has a very high RNA detection efficiency, but many cells display no RNA transcripts as only a subset of cell types is targeted. The second dataset is from a recent study of human embryonic lung mapping 147 genes in 2D^25^. The HybISS approach used for this dataset has a lower capture rate^15^ but enables the visualization of many more genes.

For such experimental data, no ground truth is available. In tissue with complex morphology such as lung, having no ground truth makes it particularly challenging to assess the method’s quality. An existing validation strategy is based on the gene expression correlation between the overlap region provided by different methods and the non-overlapping regions^15,19^. However, this validation does not compare with respect to a ground truth. Errors in existing methods could thus be propagated to the next generation of methods. Moreover, the method implicitly assumes a homogeneous spatial distribution of RNAs, including for the nuclear region. In the absence of direct access to ground truth for these imaging datasets, we chose to leverage available scRNA-seq datasets obtained from identical organ samples. These scRNA-seq datasets serve as a means to assess the consistency of single-cell spatial RNA profiling. As for simulations, we calculate the cosine distance between cell expression vectors derived from images and the nearest scRNA-seq cluster centroid. Consequently, the cosine distance between single-cell RNA profiles from the image dataset and scRNA-seq clusters serves as a surrogate measure for the quality of single-cell spatial RNA profiling.

**Fig. 3:**
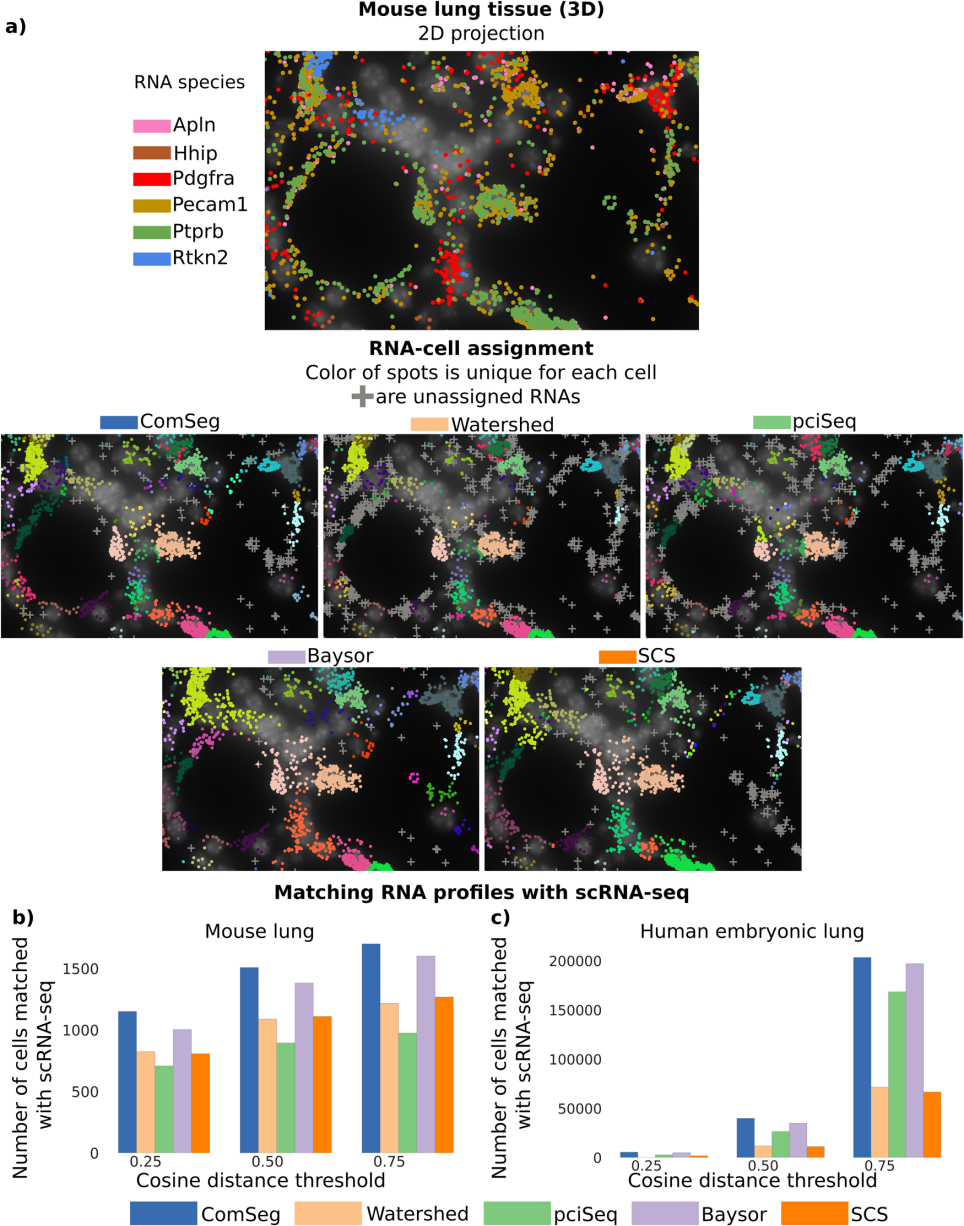
Benchmark on experimental data without cell membrane staining. **a** Mouse lung tissue and RNA assignment of the benchmarked models. **b**, **c** Number of single-cell RNA profiles identified in images matched with a scRNA-seq reference dataset at different cosine distance thresholds for mouse lung tissue (**b**) and human embryonic lung tissue (**c**).

Similarly to what we did with simulations, we applied SCS, Baysor, pciSeq, Watershed and, ComSeg on the 3D mouse lung tissue dataset (Fig. 3a) and on the 2D embryonic lung tissue dataset.

On mouse lung tissue, ComSeg identifies more cells than the other tested methods, for which an RNA profile can be assigned (Supplementary Fig. 3). We obtain similar results for human embryonic lung tissue (Supplementary Fig. 3) where ComSeg also detects more cells than the four other methods we tested. We took into account only cells with more than five RNAs. Importantly, the number of cells with matching expression profiles in the scRNA-seq data is higher for ComSeg both for mouse (Fig. 3b) and human lung tissue (Fig. 3c) at the different cosine distance thresholds.

In summary, ComSeg detects more cells than other methods, and our analysis revealed that the RNA profiles measured in the segmented cells also better fit external datasets, thus suggesting better segmentation quality.

### Application to experimental data with cell membrane staining

In order to investigate the performance of experimental data, we applied ComSeg to two publicly available MERFISH datasets, containing both DAPI and membrane staining. While the cell membrane staining does not cover all cells, it provides a valuable ground truth in some parts of the image.

**Fig. 4:**
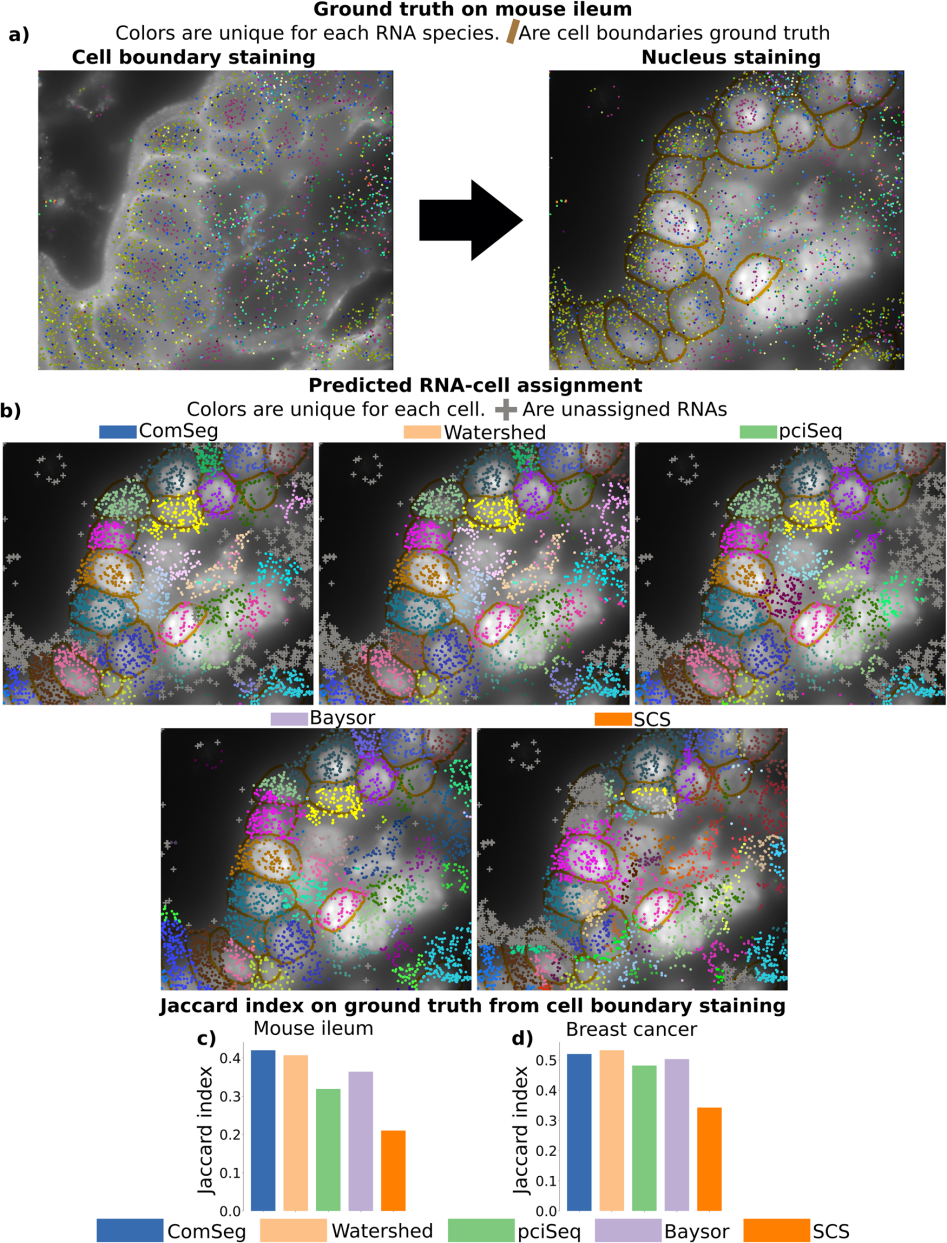
Benchmark on experimental data with partial cell membrane staining. **a** Mouse ileum tissue with Membrane staining (right) is employed to establish a segmentation ground truth (left). **b** RNA assignment of the benchmarked models on the mouse ileum dataset. **c**, **d** Jaccard index on the established ground truth from membrane staining for benchmarked models on mouse ileum tissue (**c**) and human breast cancer tissue (**d**).

The first dataset comprises a mouse ileum tissue section measuring 400 × 600 μm^15^ with 241 genes. This dataset presents challenges, as mouse ileum is known to contain RNAs with preferential intracellular distributions (Fig. 4a and Supplementary Fig. 4). The authors of this dataset leveraged a pan-cell-type cell surface marker to visualize the cell membrane. However, this surface marker tends to work more effectively in specific locations and cell types, as evidenced in Fig. 4a, where the membrane staining is more pronounced on the tissue periphery.

In addition, we applied the benchmarked methods to another MERFISH dataset featuring human breast cancer tissue. This dataset is part of the publicly released *MERSCOPE FFPE Human Immuno-Oncology Data Release* by the Vizgen company. It encompasses 550 genes spatially resolved over a section of approximately 10 mm × 8 mm. For the purpose of this study, we analyzed a 2D subsection measuring 1900 × 970 μm. Similarly to the previous dataset, it includes nuclei staining and membrane staining.

These two datasets exhibit partial membrane staining, which we used as annotated ground truth to evaluate the performance of the benchmarked method. To ensure a substantial number of annotated cells, we automated the annotation process as follows: first, we independently segmented the nuclei and the cell membrane using Cellpose^35^. Then we defined as ground truth the cells containing exactly one nucleus, thus removing cell segmentation errors likely to correspond to artifacts (e.g., visual patterns in the image that Cellpose erroneously detected as cells) and segmentation errors due to low-quality membrane stain, resulting in multiple nuclei per cell (e.g., by missing the separating membrane). As a result, we obtained a high-confidence ground truth segmentation map (see Methods for details). The ground truth segmentation maps used in this study, as well as the corresponding segmentation map from the benchmarked methods, can be found at (https://zenodo.org/records/11237477). An example of the annotations generated using this process is depicted in Fig. 4a.

We used these high-confidence datasets to evaluate the performance of the benchmarked methods. We first associated each annotated cell with its best match from each benchmarked method (Fig. 4b). Subsequently, we calculated the Jaccard index on the RNA set. On the mouse ileum dataset, Watershed and ComSeg performed slightly better than pciSeq and Baysor with a Jaccard index of around 0.4 against 0.36 and 0.31 for Baysor and pciSeq (Fig. 4c). In the breast cancer dataset, Baysor, pciSeq, Watershed, and ComSeg yield comparable Jaccard index, around 0.5 (Fig. 4d). Across both datasets, SCS exhibited a lower Jaccard index than the other methods, potentially due to the fewer resolved RNA species compared to the original study and the need for SCS to lower spatial resolution to be applied to IST data. Besides, the Jaccard index metric can hide disparities in terms of segmentation error as shown in the simulation section.

In summary, this last section shows that ComSeg can handle datasets with a high number of genes and with RNA with preferential intracellular distribution like in the mouse ileum dataset (Supplementary Fig. 4).

## Discussion

Imaging-based spatial RNA profiling methods provide RNA point cloud coordinates without information about the cell of origin of each molecule. Still, it is essential to correctly assign RNAs to cells to perform downstream tasks at the single-cell level such as cell type calling or inference of cell interaction. In this study, we present a method called ComSeg, a graph-based method operating directly on the RNA coordinates and nucleus positions. In addition, ComSeg is cell shape agnostic which makes it particularly useful for complex tissues, where cell shape convexity cannot be assumed. As such, ComSeg is a flexible method able to handle various situations, demonstrating good performances even in challenging experimental settings.

In order to compare ComSeg to other methods, we have developed a simulation framework (*SimTissue*) that provides tissue architectures which are generated from real images and are therefore reasonably realistic. Our simulation environment also encompasses simple geometric shapes which are ideally suited to study failure modes and limitations of algorithms thanks to the simplified geometry. Indeed, quality assessment is a critical aspect of segmentation, but actually difficult to perform. So far, there are no manually annotated ground truth datasets for imaging-based spatial RNA profiling. In this study, we use an automated workflow to create ground-truth data for cells with high-quality membrane stains. However, considering solely these cells for assessing spatial RNA profiling performance could introduce bias as membrane staining may work more effectively in specific locations of the tissue or only for certain cell types. Comparison to a consensus is an option that has been adopted by several authors^15,19^. We argue that this can lead to the propagation of systematic errors. For instance, all competing methods rely on shape priors that are not always met in complex tissues. A comparison to the consensus would not be able to reveal such systematic errors. Furthermore, in most cases, the consensus region will contain the nuclear region, and it is possible that the expression profile measured in the nuclear region is different from peripheral regions in the cell.

We found that most methods have difficulties with non-convex shapes and large differences in gene expression density. Our simulations suggest that error rates in cell-type calling are non-negligible: as much as 26% of cell-type assignments are erroneous because of segmentation errors when using previously published methods. ComSeg outperforms competing methods by a large margin on complex tissues. However, even with ComSeg, cell classification errors due to wrong segmentations amount to 14%, thus suggesting that the development of novel segmentation methods remains an important topic for imaging-based spatial RNA profiling data. Besides, as the number of methods and datasets continues to increase, finding optimal parameters for each of them to obtain fair comparison becomes increasingly challenging. We believe that independent benchmark studies for spatial RNA profiling data would be beneficial for the community.

Beyond these benchmark results, ComSeg has several other compelling aspects that make it interesting for the scientific community. First, it does not require membrane stainings. Indeed, existing membrane stainings are highly variable and not very robust. Moreover, they are inhomogeneous and can, therefore, lead to spatial biases in cell-type calling accuracies. In this context, methods like ComSeg, directly operating on RNA point clouds, complement cell membrane staining-based approaches like Cellpose, in cases where cell membrane staining is unfeasible, e.g., in regions of low staining quality or for certain cell types. Second, ComSeg does not require external datasets, such as scRNA-seq, which makes it also applicable in small-scale studies, where such data is not available. Moreover, ComSeg does not rely on annotated data, which is very tedious and sometimes impossible to provide. Its modular structure makes it easy to tailor ComSeg to particularities in the datasets. Lastly, ComSeg demands only basic desktop computational resources and exhibits reasonable runtime and memory requirements (see Supplementary Table 1).

One of the limitations of ComSeg is that it is dependent on the choice of cell type marker genes. The model may fail if the spatially resolved RNA species are not discriminative of cell type or cell state. A potential improvement of ComSeg would be to incorporate in the model several landmark stainings. Another potential enhancement could be to include more spatial information in the clustering of the RNA communities expression profiles. In ComSeg, RNA communities with similar expression profiles are grouped into clusters using the Leiden algorithm which leverages solely expression profiles. However recent clustering methods for spatial transcriptomic were proposed leveraging both spatial information and expression profile^36,37^. For instance, if an RNA community with a given transcriptomic profile is erroneously linked to another profile, this mistake could potentially be corrected by taking neighboring profiles into account. For instance, if the RNA community is surrounded by other communities from the same correct transcriptomic profile, a wrong assignment could be avoided. Finally, ComSeg relies on the position of single RNA molecules which prevents its application on sequencing-based spatial transcriptomic data with low spatial resolution. Indeed, the edge weights are defined with gene co-expression as similarity metrics. Applying ComSeg on sequencing-based data with low spatial resolution would require defining an expression vector similarity metric between binned spots which might be an interesting extension. Finally, ComSeg could, in principle, be used on sequencing-based data if the spatial resolution is close to IST, such as Stereo-seq^14^.

Altogether, we believe that this model will be of great interest to the community and has the potential to overcome current shortcomings in cell segmentation and cell type calling from spatial RNA profiling data. To facilitate the use of ComSeg, we have made it available as an open-source and documented Python package: https://github.com/fish-quant/ComSeg. We also make the simulation framework S*imTissue* (https://github.com/tdefa/SimTissue) available to the community, which might help researchers in the future to benchmark their methods.

## Material and methods

### Description of ComSeg algorithm

We assume that we have for each cell its centroid. In practice, this cell centroid is inferred from the nuclear stain, which are available in virtually all IST datasets. ComSeg associates the detected RNA molecules with their corresponding cell centroid. It leverages a k-nearest neighbor (KNN) graph, where the nodes are the detected RNAs. The method can be decomposed into five steps:

1. Computation of a proximity-weighted expression matrix
2. Construction of KNN graph weighted by co-expression
3. Graph community detection using the cell nucleus.
4. Cell segmentation-free in situ clustering of communities
5. Final RNA assignment

The only hyper-parameters exposed to the user are the mean cell diameter *D* and the maximum cell radius *R*_max_. The numerical values of these hyper-parameters are detailed in Supplementary Note 1.

### Proximity-weighted expression matrix

ComSeg leverages gene co-expression information. In principle, co-expression information can originate from parallel scRNA-seq data or from other published resources. However, such external datasets are not always available for the biological system under study. Hence, we estimate co-expression using only the spatial arrangements of detected RNA molecules in the image. For this, we leverage the spatial correlation between the different RNA species molecules as a proxy for gene co-expression.

For each RNA position *x*, we define a local proximity-weighted expression vector (PE) *V(x)*. For this, we consider a maximum of *K* *=* 40 neighbors *y*, positioned at a maximal distance of ${{{{{{\boldsymbol{R}}}}}}}_{{{{{{\boldsymbol{PE}}}}}}}{{{{{\boldsymbol{=}}}}}}{{{{{\boldsymbol{D}}}}}}{{{{{\boldsymbol{/}}}}}}{{{{{\boldsymbol{2}}}}}}$ from *x*. A subset of these neighbors are transcripts of the gene *g*. We thus define a local expression score of gene *g* in proximity of *x*:

1 $${V}_{g}\left(x\right)={\varSigma }_{y\in {KNN}(x),{gene}(y)=g}\,({{{{{{\boldsymbol{R}}}}}}}_{{{{{{\boldsymbol{PE}}}}}}}-{{{{{\rm{||}}}}}}x-y{{{{{\rm{||}}}}}})/{R}_{{PE}}$$

We note that each transcript at position *y* is weighted by a score that linearly decreases between *x* and the circle around *x* with radius ***R***_***PE***_. The underlying idea is that close transcripts should contribute more to the local expression estimation.

The local proximity-weighted expression vector V(x) is then defined as:

2 $$V\left(x\right)=[{V}_{g1}(x),{V}_{g2}(x),\,...,\,{V}_{{gN}}(x)]$$

Of note, the proposed PE are similar to the Neighborhood Composition Vectors proposed in ref. ^15^ with the difference that it weights RNA positions by a distance score, such that closer RNAs contribute more than RNAs that are far.

By stacking the *V(x)* for all positions *x*, we get a proximity-weighted expression matrix $V\in {{\mathbb{R}}}^{{N}_{x}\times {N}_{g}}$ where *N*_x_ is the number of detected RNA and *N*_*g*_ the number of marker genes. From this, we can finally compute the co-expression matrix $W\in {{\mathbb{R}}}^{{N}_{g}\times {N}_{g}}$, where each element ${w}_{i,j}$ is the Pearson correlation of the columns of *V* corresponding to the expression of genes *i* and *j*:

3 $${w}_{i,j}={Corr}({V}_{i,}{V}_{j})$$

The computation of these co-expression values is implemented by the Python class *ComSegDataset* of our package. Of note, the co-expressions could alternatively be computed from external data, e.g., from single-cell RNA sequencing data.

### KNN graph weighted by co-expression

Our algorithm operates on a weighted KNN graph, where the RNA molecules (across all genes) are the nodes (*K* = 10 and the max edge distance ***R***_***knn***_ between molecules is set to ***D/4***) and the weights are the estimated co-expression values ${w}_{i,j}$ defined in (3): edges between RNA molecules from strongly co-expressed genes obtain a large weight, while RNA molecules from genes that are not co-expressed are assigned a small weight. The rationale is that RNAs from co-expressed genes are likely to belong to the same cell, while RNA molecules from genes that are usually not expressed together are more likely to belong to different cells.

It is worth mentioning that we opted to keep the value of *K* fixed at K = 10. Indeed, modifying this parameter has a negligible impact on performance, as illustrated in Supplementary Fig. 5.

### Graph community detection algorithm

In the previous section, we generated a graph strongly connecting RNA nodes that are likely to belong to the same cell. Now, our objective is to partition this graph into sets of RNAs belonging to the same cells. To achieve this, we developed a modified version of the Louvain method^27^ for community detection so it can accommodate prior knowledge given by nuclei segmentation or other landmarks.

The original Louvain algorithm is a widely used method to partition a graph into sets of strongly connected nodes. These strongly connected sets of nodes are called communities. The algorithm optimizes a metric of graph structure called the modularity, noted *Q*^38^. *Q* is the sum of the differences between intra-community weights and their expected value in a randomly rewired graph. *Q* can be expressed as a sum over the edges *(u,v)* of the graph.

4 $$Q=\frac{1}{2m}{\varSigma }_{u,v}\left[{w}_{{g}_{u},{g}_{v}}-\frac{{k}_{u}{k}_{v}}{2m}\right]\delta ({C}_{u},{C}_{v})$$

Where *g*_u_ and *g*_*v*_ are the gene index of the RNA nodes *u* and *v*. ${w}_{{g}_{u},{g}_{v}}$ is the corresponding weight from the co-expression matrix *W. k*_*u*_ is the degree of node *u* defined as ${{k}_{u}=\varSigma }_{v}{w}_{{g}_{u},{g}_{v}}$, m is the sum of the weights in the network $m={\frac{1}{2}\varSigma }_{u,v}{w}_{{g}_{u},{g}_{v}}$ and the function *δ* is 1 if the nodes *u* and *v* belong to the same community *C* (i.e., *C*_*u*_ = *C*_*v*_) and 0 otherwise.

This method iterates two elementary phases: in the first step, modularity *Q* is greedily optimized. For this, we start from an initialization where each node is assigned to its own community. Nodes are then moved in a random order to neighboring communities to greedily maximize modularity. This first step stops when no node move improves the modularity *Q*. The randomness of this phase has a negligible effect on the final ComSeg output as studied in Supplementary Note 3.

In the second step, a new aggregated network is built where communities found in the first step become nodes. Edge weights between those new nodes are calculated as the sum of the edge weights between the identified communities. We can then re-apply the first step on this aggregated network until there is no modularity gain.

When applying the Louvain method, we only consider positively weighted edges as negatively co-expressed genes are not likely to belong to the same cell. Besides, in order to introduce prior knowledge in the form of landmark segmentation, we modify the method as follows: before running the community detection method, RNA nodes inside the same segmented nucleus or chosen cell landmark are merged together into one node, which we refer to as “cell nodes”. When we apply the Louvain method, different cell nodes cannot be merged together. During the first step of local moving of nodes, nodes take the cell label of the community they are assigned to.

Of note, in most cases, nuclear staining is available, and this therefore represents the most frequent use case. However, the landmarks can also originate from other stainings (such as membrane staining).

The input graph strongly connects RNAs that are likely to belong to the same cell. Hence the resulting community partitions of RNAs are supposed to form sets of RNAs belonging to only one cell. In contrast, a cell might contain several communities.

The graph construction and partitioning are implemented in the Python class *ComSegGraph* of our Python package.

### Cell segmentation-free in situ clustering

In the previous step, we split our graph into communities of RNAs that are supposed to belong to the same cell. Hence, while a cell can be composed of several RNA communities, we assume that each community does not extend beyond cytoplasmic boundaries. This mimics the mechanism of superpixel segmentation in computer vision^39^.

To ease the identification of RNA communities that may belong to the same cell, we first identify communities that have similar RNA profiles.

We associate with each community *C* of RNA, an expression vector *V*_*C*_.

5 $${V}_{C}={\frac{1}{{{{{{\rm{\#}}}}}}C}}\varSigma _{x\in C}V(x)$$

where *V(x)* are proximity-weighted expression vectors (PE) computed as defined above. Summing the PE of each RNA node in the community helps to capture the local transcriptomic information missing in the global co-expression matrix *W*. Hence, each community is associated with a community expression vector *V*_*C*_ composed of co-expressed genes at the global scale but also containing the local transcriptomic information of the cell it belongs to.

Then, similarly to what is commonly done in scRNA-seq analysis^40^, we cluster our set of community expression vectors *V*_*C*_ using optionally PCA for dimensionality reduction (depending on the number of marker genes) and the modularity-based algorithm Leiden^28^. It defines community clusters $\{{L}_{i}\}$ that exhibit similar expression profiles. Each community (and each member *x*) thus receives a community profile label *L*_*i*_. However, we do not cluster community expression vectors of less than three RNAs as they might be too small to reliably capture their local transcriptomic neighborhood. We assign to these small communities the majority label of the *K* nearest neighbors.

This step thus provides a map of RNAs labeled with their community profile label *L*_*i*_. We refer to this map of labeled RNAs as the transcriptomic domain map. The in situ clustering step is implemented in the class *InSituClustering* of our Python package.

Of note, previous studies have utilized KNN graphs to investigate transcriptomic similarity^41–43^. These studies aimed to identify similarities in gene expression profiles between individual cells or groups of cells (spots). However, our method differs from these approaches in that we seek to uncover similarities between groups of RNA molecules that are not cells but represent subsets of cells.

### Final RNA assignment

In the final step, the goal is to associate the RNAs with the cell they belong to. First, we add a centroid node, a node that does not correspond to an RNA molecule. If the landmarks contain nuclear staining, this centroid node is the centroid of the nucleus, all nuclear RNAs are merged into this node, and the centroid node gets the community profile *L*_*i*_ of the nuclear RNAs. In case there is no nuclear RNA, the community profile of the centroid is defined as the majority profile among the *K* nearest neighbors (*K* = 15, **R** **=** **D/ 2**). In case there is no nuclear staining, the centroid node can be defined based on other landmarks (e.g., the maximum distance function of a cellular landmark). Cell centroid nodes are required to estimate the single-cell RNA profiles.

Once every cell centroid is associated with a community profile *L*_*i*_, we can finally estimate the single-cell RNA profiles. We associate each cell centroid of label *L*_*i*_ to their nearest RNAs of the same label. We employ the geodesic distances between cell centroids and RNA nodes. The geodesic distance is the graph’s shortest path distance between the centroids and RNA nodes and is computed with the Dijkstra algorithm^44^. We apply the Dijkstra algorithm with Euclidean distance weight on the edges. Besides, when an RNA node’s geodesic distance to its nearest centroid is superior to a chosen maximum cell radius *R*_max_, the RNA molecule is not associated with any cell. Using the geodesic distance helps to better estimate the cell size of non-convex cells when applying the cell radius *R*_max_ cut-off. Moreover, geodesic distance accommodates potential lacunar spaces within the tissue when calculating the centroid-RNA distance.

In summary, our method treats RNA positions as nodes in a graph with edges weighted by co-expression. This permits an accurate separation of cells with different expression profiles without the need for explicit cell segmentation. Our method can further use landmarks, such as nuclei, to initiate community detection in spatial RNA graphs. In the absence of clear differences in expression profiles, cells will then be separated based on RNA-centroid distance in the graph.

Our method does not use an explicit cell shape prior other than mean cell diameter *D* and maximal cell radius *R*_max_ as the goal is to make this method suitable for tissues harboring arbitrary cell shapes. Furthermore, ComSeg is not based on machine learning, and does therefore not need annotated data or time-consuming learning steps; which simplifies its application and interpretability.

### Simulation Python package

In order to validate our method and perform quantitative benchmarks against other approaches, we designed a simulation framework *SimTissue* capable of creating ground-truth data with tunable complexity for tissue morphology, cell type compositions, and marker-gene expression levels.

Our simulation framework can be divided into two steps:

1. Simulation of tissue morphology.
2. Simulation of RNA composition and spatial distribution.

*SimTissue* is implemented in Python and available at https://github.com/tdefa/SimTissue and documented at https://simtissue.readthedocs.io.

### Simulation of tissue morphology

Our framework offers two possible simulation scenarios. In the first scenario, we consider regular geometric patterns. While these are not realistic scenarios, they are well suited to point to potential problems and limitations of the algorithms. They can thus be seen as a purely methodological test scenario. Examples include the checkerboard arrangement or the simulation of clamped L-shapes with random nuclei positions.

In the second scenario, we consider more realistic tissue simulations. For this, *SimTissue* takes as input segmented nuclei from experimental data. These segmentation masks can often be generated easily from DAPI or other nuclei stainings and are widely used for image-based spatial transcriptomic experiments^8,13,33^. Individual cytoplasms are defined by growing cells from segmented nuclei. Each cell grows at a random speed to add irregularity to the cell size. Still, some organs, such as lung tissue, contain lacunar space without cells. Therefore, optional masks can be added to indicate these lacunar spaces where cells cannot grow into.

In experimental data, the tissue section is cut at an arbitrary location and some nuclei are removed from the rest of the cell. It is, hence, possible to simulate cells without nuclei to better mimic experimental fluorescent-based experiments.

### Simulation of RNA composition and spatial distribution

Once the nuclei positions and cell shapes are simulated, we have to simulate the RNA composition and distribution within each cell. RNA expression levels can either be set as constant or be directly sampled from experimental measures, e.g. from scRNA-seq data. When sampling profiles from scRNA-seq, we sample for each simulated cell an expression profile of a single cell from scRNA-seq, then multiply the number of RNAs by a factor. Here, we choose a factor of 3 as the fraction of mRNA transcripts captured per cell in scRNA-seq data can reach 30% (depending on reagent chemistry)^45^ while the capture rate of smFISH experiment is close to 100%^46^. Finally, RNA molecules are randomly positioned in the available space of the cell with a uniform spatial distribution.

In summary, *SimTissue* allows to simulate experiments of incremental complexity. The full control over the ground truth and the difficulty of the segmentation task aims to understand the limitations of the benchmarked methods.

### Simulations in this article

#### Regular pattern simulations

We simulated 2D square grids (15 μm × 15 μm) and nested L-shape patterns (four squares of 15 μm  × 15 μm). The pixel size is 0.150 µm and thus similar to the pixel size obtained with a 60x objective. Nuclei are spheres of 3.75 μm rays. Nuclei are in the center of the cell for square cell shape. For an L-shaped cell, the nucleus is randomly positioned in the center of one of the four squares composing the L-shaped cell.

#### Lung tissue simulations

We simulated 3D mouse lung tissue leveraging experimental FISH data from ref. ^33^. The original data were composed of images of 112 µm × 150 μm in XY and 15 μm in Z with a DAPI staining and a Cy3 fluorescent channel. The original pixel size is 0.103 × 0.103 µm and the Z spacing is 0.300 µm. The positions of the nuclei in our simulation are the positions of the segmented nuclei in the original images. The nuclei were segmented with Cellpose^35^ on DAPI staining.

Next, we identified the space occupied by cells by thresholding the Cy3 FISH signal (first quintile of the Cy3 distribution). Individual cytoplasms were defined by growing cells with random irregular speed from segmented nuclei inside the allowed space. Random growth speed allows to add irregularity in the cell size. Finally, we removed 20% of nuclei in our simulation to simulate nuclei missed during sample preparation, as explained above.

We used a list of 34 cell-type marker genes with the objective to classify 19 different cell types present in mouse lung tissue. The list of marker genes was selected using the NS-forest algorithm^34^ on our external scRNA-seq dataset of mouse lung tissue^33^. Then, we associated each individual cytoplasm with an expression vector sampled from our scRNA-seq dataset so that all the RNA profiles in our simulation are taken from real experimental data. This dataset can be found at https://zenodo.org/records/10172316.

### Statistics and reproducibility

We benchmark ComSeg against pciSeq^16^, Baysor^15^, SCS^19^, and Watershed method on both simulation and experimental data. These are among the frequently cited and most widely used methods today if membrane markers are absent. The hyperparameter setting of the benchmarked methods can be found in the Supplementary Note 1. The details of the metrics and datasets that were used are described in the following sections.

### Benchmarking on simulations

To assess the RNA profiling quality we compute the mean Jaccard index as follows:

For each cell *c*, ${J}_{c}=\frac{|{X}_{c}\cap {Y}_{c}|}{|{X}_{c}\cup {Y}_{c}|}$ where *X*_*c*_ is the ground truth set of RNAs associated with the cell *c* (ground truth) and *Y*_*c*_ is the set of RNAs predicted as associated with the cell *c*. The final mean Jaccard index per cell is:

6 $${J=\frac{1}{{{{{{\rm{\#}}}}}}\{c\}}}\varSigma _{c}{J}_{c}$$

For each cell *c*, we compute the percentage of wrongly associated RNA $W{A}_{c}$ as follows (False Discovery Rate):

7 $$W{A}_{c}=\frac{{{{{{\rm{|}}}}}}{Y}_{c}{{{{{\rm{\backslash }}}}}}{X}_{c}{{{{{\rm{|}}}}}}}{{{{{{\rm{|}}}}}}{Y}_{c}{{{{{\rm{|}}}}}}}$$

We compute the percentage of missing RNA $M{S}_{c}$ (False Negative Rate) per cell as follows:

8 $$M{S}_{c}=\frac{{{{{{{\rm{|}}}}}}X}_{c}{{{{{\rm{\backslash }}}}}}{Y}_{c}{{{{{\rm{|}}}}}}}{{{{{{{\rm{|}}}}}}X}_{c}{{{{{\rm{|}}}}}}}$$

To perform cell type calling from the cell expression vector from Baysor, Watershed, SCS, and Comseg, we first normalize the count matrix from both scRNA-seq and from RNA-cell association using the scTransform normalization^47^. Then we compute the cosine distance between the cell expression vector and the cell type median centroid defined in the reference scRNA-seq data from^33^. Cells are classified into their nearest cell type cluster in terms of cosine distance. For lung tissue simulation, we also employ this cell-type calling method on the ground truth expression vector of each cell to assess the maximum accuracy achievable with the 34 selected markers. Conversely, to previously cited methods, pciSeq performs cell type classification and RNA-nuclei association simultaneously. For this reason, the cell type classification described here was not applied to pciSeq.

### Experimental evaluation

We applied the benchmarked methods on two datasets of lung tissue. The first one exhibits 6 different marker genes in 3D mouse lung tissue acquired with a home-built sequential smFISH system. This mouse lung tissue was irradiated with 17 gy 5 months prior to mouse sacrifice as described in ref. ^33^. The second dataset was acquired with HybISS^26^, consisting of a human embryonic lung and 147 genes in 2D^25^. In both cases, we perform a nucleus segmentation with Cellpose^35^ and use this segmentation as initialization in our benchmark.

### Reference scRNA-seq cluster

As we do not have ground truth for all experimental data, we leverage scRNA-seq to check the consistency of the single-cell spatial RNA profiles obtained from images. We argue that it should be possible to match the spatial profiles obtained from images to the scRNA-seq data, and that the percentage of cells that can be matched is thus a quality metric for the segmentation method.

### Reference clusters from scRNA-seq for mouse lung tissue

We re-cluster our single-cell data^33^ using only the six mapped genes and cells from the same condition (i.e., 5 months after irradiation with 17 gy). The final clustering is composed of five different clusters.

### Reference clusters from scRNA-seq for embryonic lung tissue

In the original study^25^, the authors applied pciSeq to perform single-cell spatial RNA profiling. Their final expression matrix provided in the study displays 89 genes over the 147 genes map in the HyBISS data. We also keep the same subset of 89 genes for evaluation. We use the same reference scRNA-seq dataset and clustering annotation as provided by the authors.

### Matching in situ single-cell RNA profile and scRNA-seq

As for simulation, we normalize both scRNA-seq data and the count expression matrix from spatially resolved RNA profiling data with scTransform^47^. Many methods exist to match single-cell spatial transcriptomic data and scRNA-seq^48^. We chose to leverage the cosine distance as it is robust with respect to different capture rates among the two modalities.

Each cell expression vector was matched to the closest median centroid cluster from scRNA-seq. The cosine distance acts as a proxy for the matching quality.

### Automatic generation of ground truth for high-quality staining area

We segment the nuclei and the cell using Cellpose 2.0^49^. We manually fine-tuned the Cellpose models with the provided human-in-the-loop GUI except for the nuclei human breast cancer dataset where we kept the default cellpose nuclei model. Specifically, in the human breast cancer dataset, we segmented cells based on the third cellbound marker across three different available. For the cells in the mouse ileum dataset, we kept the fine-tuned Cellpose segmentation proposed in the original publication. After segmentation, we identified cells with exactly one nucleus. However, in dense tissue areas, cells might mistakenly include a few pixels from another nearby nucleus due to segmentation inaccuracies. In order to be robust with respect to this kind of variations, we assigned a nucleus to a cytoplasmic region if there was an overlap of at least 20-pixel width between them. The final list of cells containing exactly one nucleus is then employed to generate the ground truth, as these cells are likely to correspond to high-quality staining areas.

To compute the Jaccard index on the generated ground truth mask, we associate each cell from the ground truth with the predicted cell with the most molecules in common. We then apply the formula described in (6).

### Ethics statement

For the generation of the in-house mouse lung tissue dataset displayed in Fig. 3: studies were performed in accordance with the recommendations of the European Community (2010/63/UE) for the care and use of laboratory animals. Experimental procedures were specifically approved by the ethics committee of the Institut Curie CEEA-IC #118 (Authorization number APAFIS#5479-201605271 0291841 given by the National Authority) in compliance with the international guidelines. Females C57BL/6J mice purchased from Charles River Laboratories at the age of 6 weeks were housed in Institut Curie animal facilities.

### Supplementary information

Peer Review File

Supplementary Information

## Data Availability

The simulated dataset of lung tissue can be downloaded from Zenodo (https://zenodo.org/records/10172316). The generated ground truth of the two MerFISH datasets and the corresponding segmentation results from our benchmark are available at https://zenodo.org/records/11237477. Our in-house mouse dataset is available at https://zenodo.org/records/11068509.

## Code availability

ComSeg is available at https://github.com/fish-quant/ComSeg. The implementation code of SimTissue is available at https://github.com/tdefa/SimTissue. Scripts to reproduce the benchmark are available also on github https://github.com/tdefa/script_Benchmark and also available at this Zenodo repository^50^: 10.5281/zenodo.11505979.
