# Supplementary material for: A point cloud segmentation framework for image-based spatial transcriptomics
Source: Commun Biol. 2024 Jul 6;7:823. doi: 10.1038/s42003-024-06480-3 (PMC11227573; doi:10.1038/s42003-024-06480-3)
Supplement: Supplementary file 2 — Supplementary Information [file 42003_2024_6480_MOESM2_ESM.pdf]

Supplementary Note 1: Hyper-parameter setting of benchmarked methods

ComSeg

We apply ComSeg with mean cell diameter $D = 10\mu m$ and $R_{max} = 15\mu m$ in checkerboard simulation (Sim 1-2-3-4) and increase to $R_{max} = 40\mu m$ in Sim 5 while keeping the same value for D.

We set $D = 15\mu m$ and $R_{max} = 30\mu m$ in lung tissue simulation. We use $D = 20\mu m$ and $R_{max} = 40\mu m$ on mouse lung tissue and $D = 40\,pixels$ and $R_{max} = 50\,pixels$ on embryonic lung tissue. For the Mouse ilieum dataset we use $D = 10\mu m$ and $R_{max} = 8\mu m$. For the Breast cancer dataset we use $D = 5\mu m$ and $R_{max} = 12\mu m$.

Baysor:

We apply Baysor by incorporating nucleus segmentation mask priors and let Baysor estimate the scale parameter automatically. All other parameters were left as default. In addition, for the mouse ileum dataset, we kept the original parameters set by the authors including a compartment specific gene list as parameter, and we added the nuclei segmentation mask priors.

Baysor does not perform RNA-nucleus assignment but groups RNAs into cells without referring to the given nucleus segmentation index of the prior segmentation mask. On simulated datasets, in order to compare Baysor to other methods for RNA-nucleus assignment, we associated each predicted cell index by Baysor with a nucleus segmentation index from the provided segmentation mask ground truth. To that end, each predicted cell index by Baysor was associated with the nucleus index from the ground truth with the most molecules in common.

pciSeq

We apply pciSeq on geometric simulation using simulated scRNA-seq data containing the simulated RNA profiles. In lung tissue simulations, we use the same scRNA-seq dataset as the one sampled to simulate RNA profiles. All other parameters were left as default. We use the version 0.0.46 of the pciSeq Python PyPI package. We use maximum projection to apply pciSeq to 3D data as pciSeq is only designed for 2D.

For the MERFISH human breast cancer and MERFISH mouse ileum datasets, inspired by the original publication of the MERFISH mouse ileum dataset[1], we use the RNA in the nuclei as reference scRNA-seq data.

Watershed

We apply Watershed by taking as input the inverse distance map from segmented nuclei. In lung simulation, as a mask, we use the inferred cytoplasm from the Cy3 signal. Otherwise, we use the Watershed on the inverse distance map with a maximum distance from the nucleus of 2 µm on mouse lung tissue, of 16 pixels for embryonic lung tissue and of 8 µm on the MERFISH human breast cancer and MERFISH mouse Ileum datasets. The mask helps to prevent the misassignment of RNA to nuclei too far apart and was adapted to the tissues density and complexity

SCS

We use the SCS code released by the author (https://github.com/chenhcs/SCS/) as well as their training parameter display in their example (100 epoch and a learning rate of 0.001). To adapt SCS to image based spatial transcriptome data we use a binning of 15 pixels. Like Baysor, SCS does not perform RNA-nucleus assignment but groups RNAs into cells. Hence we use the same method as for Baysor to assess performance. We use a crop of 3000x3000 for the mouse ileum dataset and crop of 9000x9000 for the human lung and merfish breast cancer dataset. For all datasets we use Cellpose[2] to segment the nuclei instead of the originally proposed watershed.

Supplementary note 2 : Validation on simple simulations of regular patterns

In this section we aim to better understand the strengths and limitations of each benchmarked method. To this end, for validation and benchmarking, we generated five types of simulated datasets of gradually increasing complexity and specific cases.

To study the effect of the different possible expression profiles without any cell shape complexity, we simulate a checkerboard. This is a similar cell size scale to what we can observe in mouse tissue[3,4]. To test the effect of non-convex shapes, we also simulate L-shaped cells (see Methods). Each simulated set contains 10 images of 110 cells except the last one containing 144 cells per image.

1) **Simulation 1 (Sim 1). Variation of expression level:** The objective of this simulation is to benchmark the methods when markers have the same expression level versus the case where one marker is sparsely expressed. We started by simulating only two cell types expressing one marker each, A and B. We fixed the number of transcripts for the first cell type to A=100, while we set the number of transcripts for the second to B=100

(Sim1a) or B=10 (Sim1b) in two runs (Supplementary Figure 1a, left panels). For this geometry, the Watershed performs a perfect assignment, as the nuclei are centered and the cell shapes are convex. Besides, in the simple case where the two markers are equally expressed, Baysor and ComSeg have also a Jaccard index close to 1 whereas pciSeq has a Jaccard index below 0.8. In fact, pciSeq only assigns RNAs in the close neighborhood of the nucleus and with a spherical shape prior. These missed RNAs are penalized by the Jaccard index.

When the expression of the second cell type becomes sparse (B=10), the performance of all methods leveraging RNA spatial distribution, Baysor, pciSeq and ComSeg drops. Still, ComSeg performs better in terms of Jaccard index (over 0.8, Supplementary Figure 1b, center left panel) and cell type accuracy (of 0.99, Supplementary Figure 2) than Baysor and pciSeq. This result can be attributed to the utilization of a cell shape prior by Baysor and pciSeq in RNA assignment. When expression becomes sparse, Baysor and pciSeq may not find RNA point clouds matching their shape prior. Finally, the shape agnostic strategy of ComSeg appears to be more adapted for sparse input.

2) **Simulation 2 (Sim 2). Shared marker genes:** In the subsequent simulation, we aimed to investigate the impact of shared marker genes across diverse cell types. To achieve this, we categorized three distinct cell types: cell type A, with 100 RNAs from gene A; cell type B, with 100 RNAs from gene B; and cell type C with 100 RNAs from gene A and gene B (Supplementary Figure 1a, center right panel). In this case pciSeq and Baysor have slightly better performance in terms of Jaccard index than ComSeg (Supplementary Figure 1b, center right panel). Still, all models get an almost perfect cell type calling (Supplementary Figure 2). Without surprise, Watershed obtains perfect RNA-nuclei assignment due to the simplicity of the tissue geometry.
3) **Simulation 3 (Sim 3). Experimental expression profile:** In this simulation, we sample RNA profiles from experimental data. We simulate 34 marker genes as described above for lung tissue simulation (Supplementary Figure 1a, right panel). On real RNA profiles, ComSeg has a better Jaccard index than Baysor and pciSeq. As the cell shapes are still convex, Watershed gets a Jaccard index of 1 (Supplementary Figure 1b, right panel).
4) **Simulation 4 (Sim 4). Experimental expression profile with missing nuclei:** In this simulation, a scenario akin to the previous one was replicated, where some nuclei were intentionally omitted to simulate conditions akin to experimental data (Supplementary Figure 1c, left panel). Specifically, 20% of the cells were simulated without nuclei, mirroring situations encountered in tissue experiments. Remarkably, under these conditions, ComSeg continued to exhibit a superior Jaccard index compared to both Baysor and pciSeq (Supplementary Figure 1e, left panel). As expected, the Watershed method cannot cope with missing nuclei, as the method uses nuclei as seeds. As a consequence, accuracy in cell type identification drops as compared to other models (Supplementary Figure 1f, left panel). A visual representation of the benchmarked methods RNA assignments can be found in Supplementary Figure 1c.
5) **Simulation 5 (Sim 5). Experimental expression profile with missing nuclei and L shape:**

Lastly we also add cells with an L-shape to test non-convex examples (Supplementary Figure 1d, left panel). In this case, ComSeg outperforms other models for the Jaccard index confirming that it is designed to deal with irregular non-convex cell shape. Conversely, Watershed has a very low Jaccard index because it cannot cope with non-convex shapes by construction (Supplementary Figure 1e). Also, Baysor and pciSeq are underperforming in accurately identifying the L-shaped cells owing to their inherently convex cell shape assumptions. As a consequence, ComSeg also exhibits superior cell type calling performances in this more complex case as compared to all other methods (Supplementary Figure 1f). A visual representation of the RNA assignments generated by all models can be found in Supplementary Figure 1d.

In summary, all methodologies encounter difficulties as cell shapes and RNA profiles become increasingly complex and as marker expression becomes sparse. Notably, Watershed proves to be an optimal choice for cells with convex shapes. PciSeq and Baysor, while capable of estimating valid single-cell spatial RNA profiles in terms of cell type calling, exhibit limitations in capturing a substantial portion of transcripts. Moreover, the disparity in RNA-cell assignment performance between ComSeg and the other benchmarked methods widens notably as cell shapes and expression patterns grow in complexity.

Supplementary Note 3 : Influence of initialization seed on ComSeg

ComSeg employs random initialization within the Louvain method when computing the communities and the Leiden method when clustering the $\{V_C\}$. To evaluate the impact of the random initialization, we executed the algorithm 50 times on the lung simulation and compared the mean Jaccard index per cell for each run with a different random initialization (Supplementary Figure 6). The standard deviation of the mean Jaccard index per cell is less than 0.002, demonstrating the negligible influence of the initialization seed.
Similarly, we compute the Rand index between the RNA assignments over 10 runs on Supplementary Figure 7. This analysis shows that the Rand index between pairs of runs is stable with a value of approx 0.96, indicating that 96% of the RNA are grouped into a similar way across different initializations.

Supplementary figures

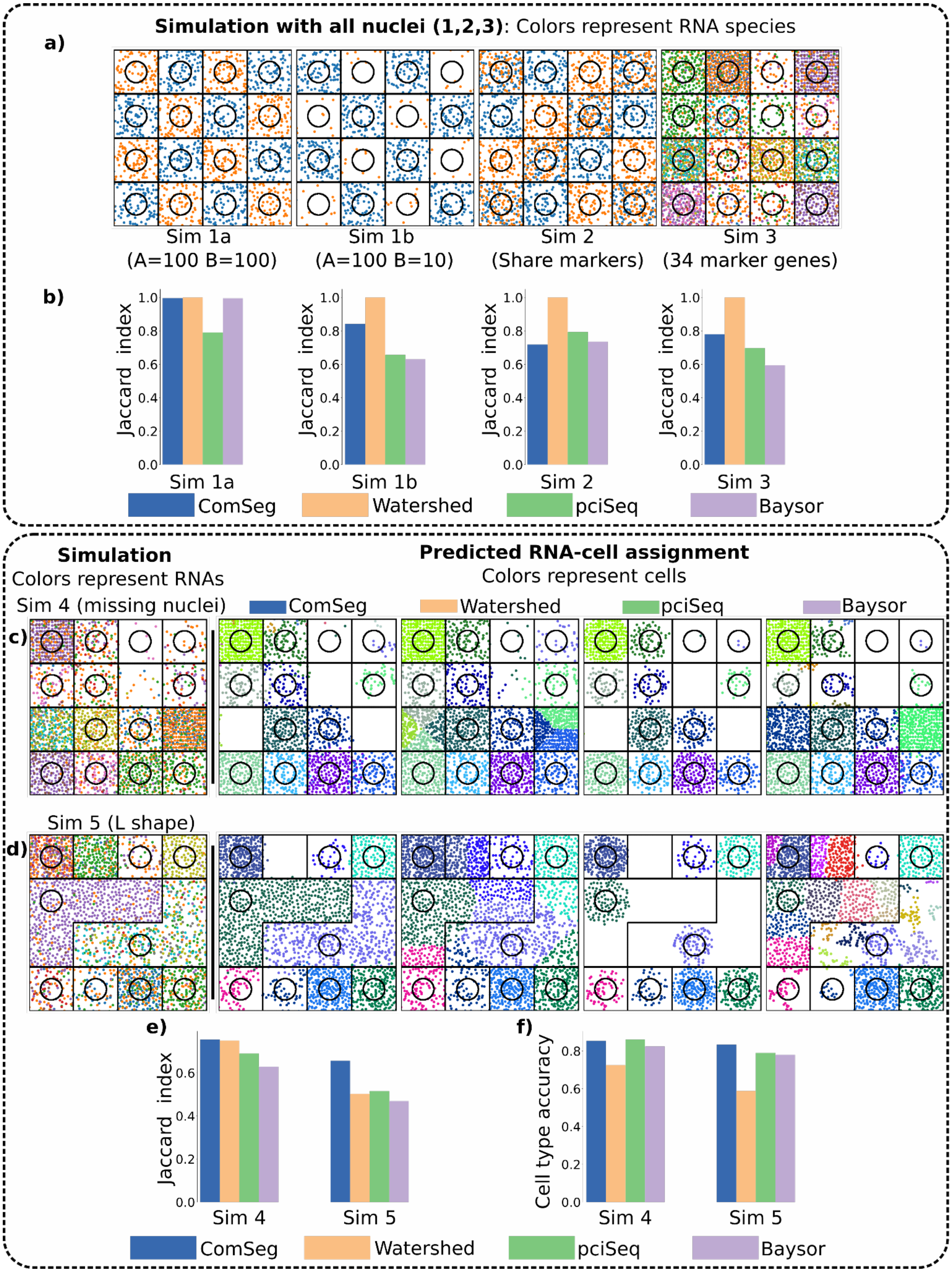

Simulation with all nuclei (1,2,3): Colors represent RNA species
a)
Sim 1a
(A=100 B=100)
Sim 1b
(A=100 B=10)
Sim 2
(Share markers)
Sim 3
(34 marker genes)
b)
Jaccard index
1.0
0.8
0.6
0.4
0.2
0.0
Sim 1a
Sim 1b
Sim 2
Sim 3
ComSeg
Watershed
pciSeq
Baysor
Simulation
Colors represent RNAs
Predicted RNA-cell assignment
Colors represent cells
Sim 4 (missing nuclei)
c)
Sim 5 (L shape)
d)
e)
Jaccard index
0.6
0.4
0.2
0.0
Sim 4
Sim 5
f)
Cell type accuracy
0.8
0.6
0.4
0.2
0.0
Sim 4
Sim 5
ComSeg
Watershed
pciSeq
Baysor

Supplementary Figure 1 : Simulations and results on regular patterns. **a** Examples of simulation where all cells have a nucleus. From left to right: Sim 1 with variation of expression level, Sim 2 with share marker genes, Sim 3 with experimental RNA profiles with 34 markers. **b** Mean Jaccard index per cell for Sim 1-2-3. **c** Example of Sim 4 with experimental RNA profiles with 34 markers and the corresponding RNA-cell assignment for the benchmarked methods**. d** Example of Sim 5 akin to Sim 4 but with L-shaped cells and the corresponding RNA-cell assignment for the benchmarked methods**. f** Mean Jaccard index per cell for sim 4 and 5**. e** Cell type calling accuracy for Sim 4 and Sim 5.

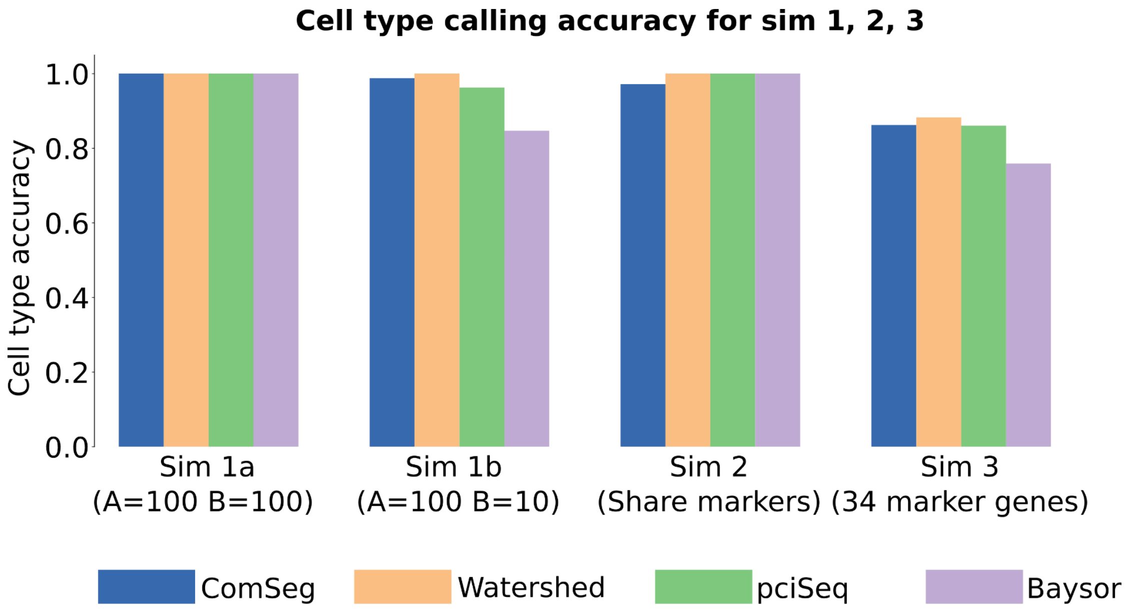

Supplementary Figure 2: Cell type calling accuracy on checkerboard cell for the Sim 1 with different levels of expressions, Sim 2 with share markers and sim 3 with 34 marker genes with RNA profiles sample from scRNA-seq.

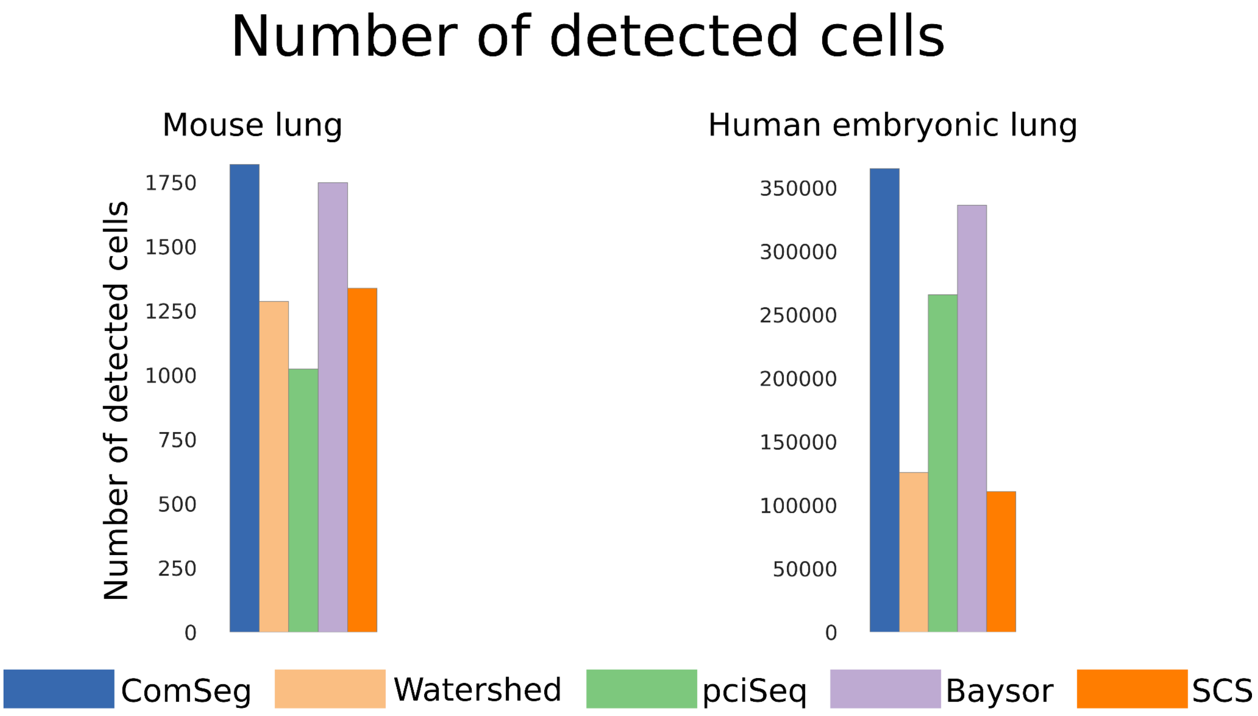

Supplementary Figure 3 : Number of cells associated with an RNA profile (more than 5 RNAs) in the mouse lung tissue dataset (right) and in the Human embryonic lung tissue dataset (Left) for the tested method.

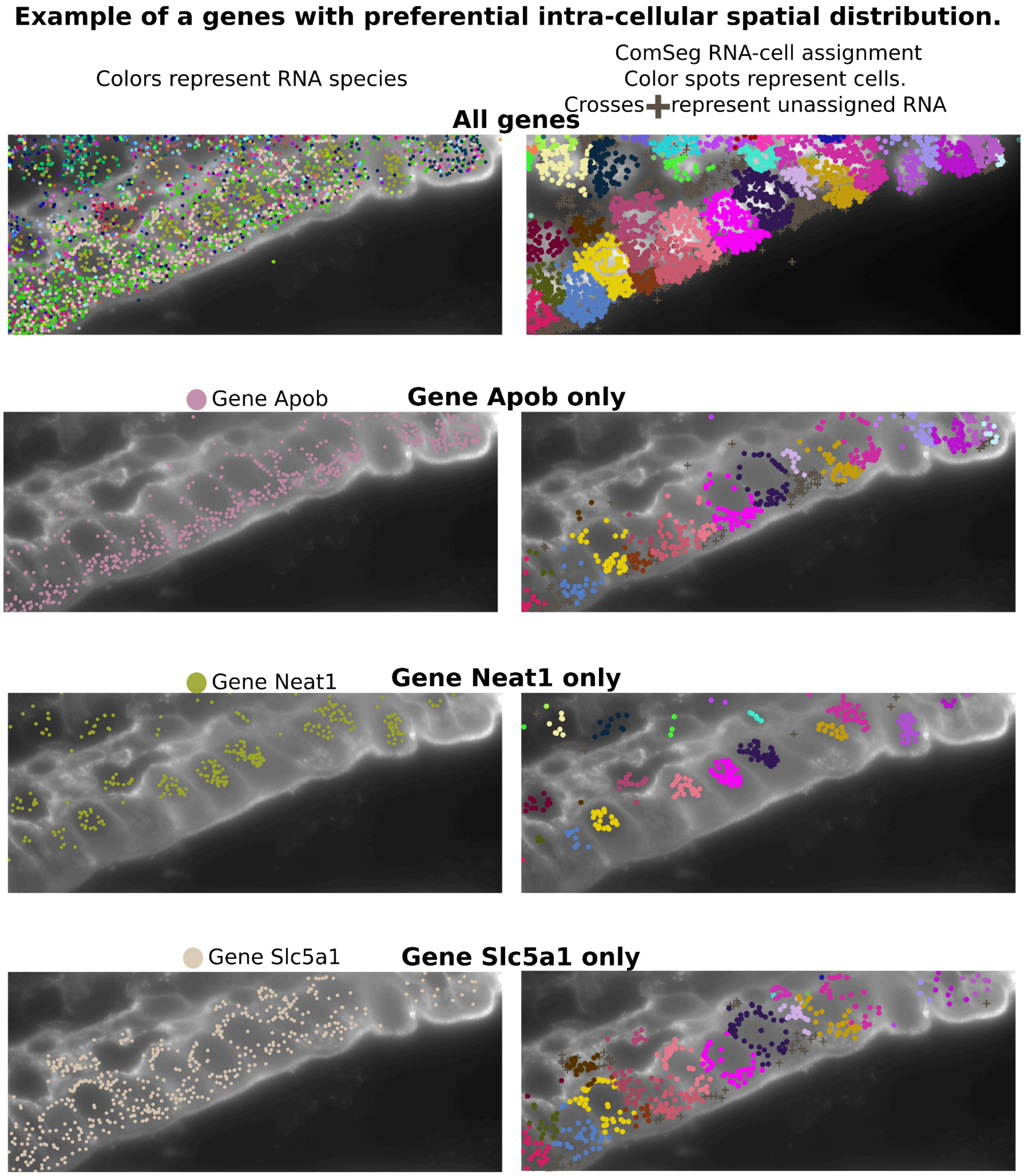

Supplementary Figure 4 : Example of genes with non uniform intracellular spatial distribution in the mouse ileum dataset. All the rows display the membrane staining in gray. The first row of images shows plots with all the genes. The second, third and fourth rows show only results with the gene Apob, Neat1 and Slc5a1 respectively displaying preferential intra-cellular spatial distribution.

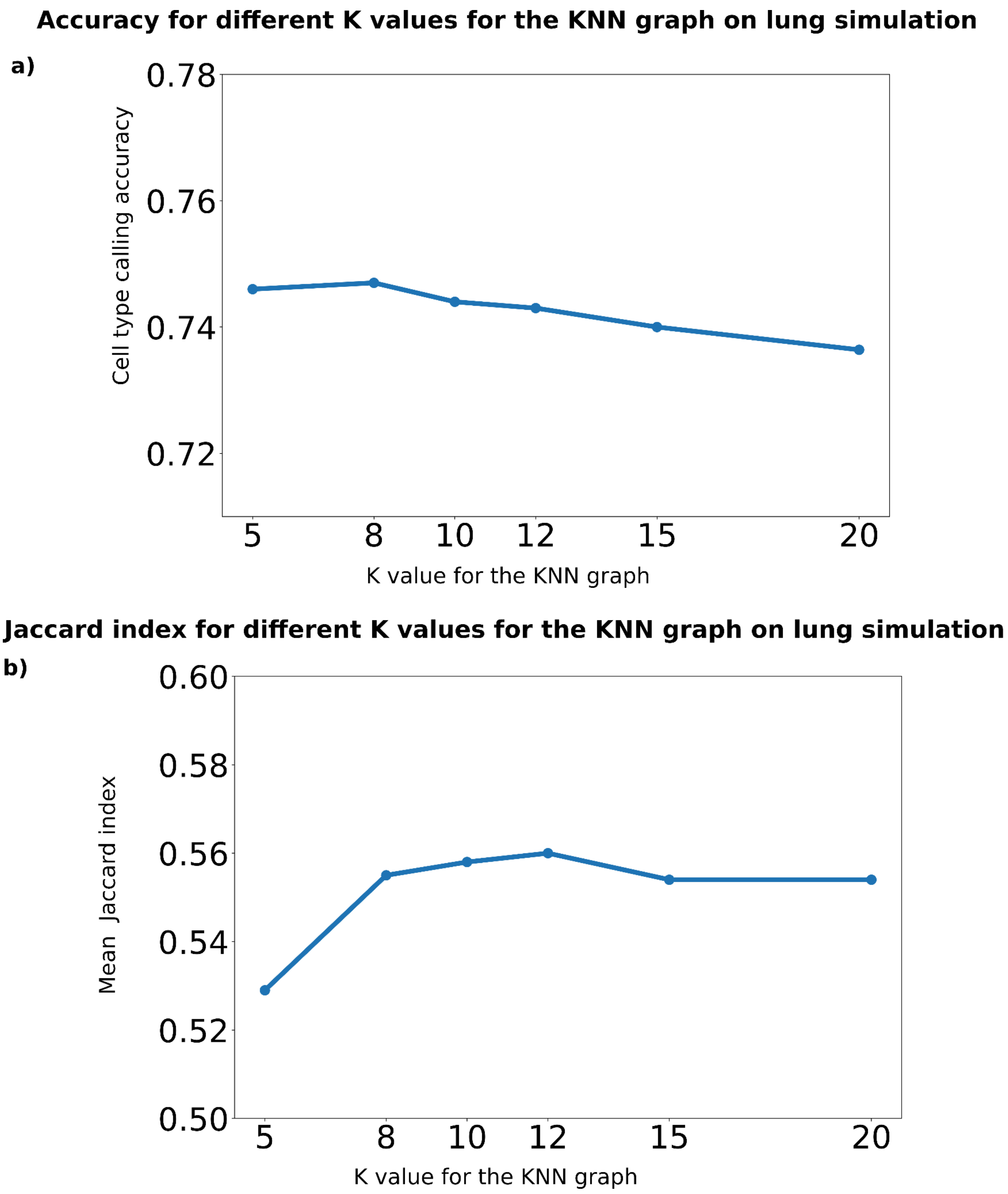

Supplementary Figure 5: Performance of ComSeg with different K values for generating the KNN graph on lung simulation. We measure the cell type calling accuracy (**a**) and the Jaccard index (**b**).

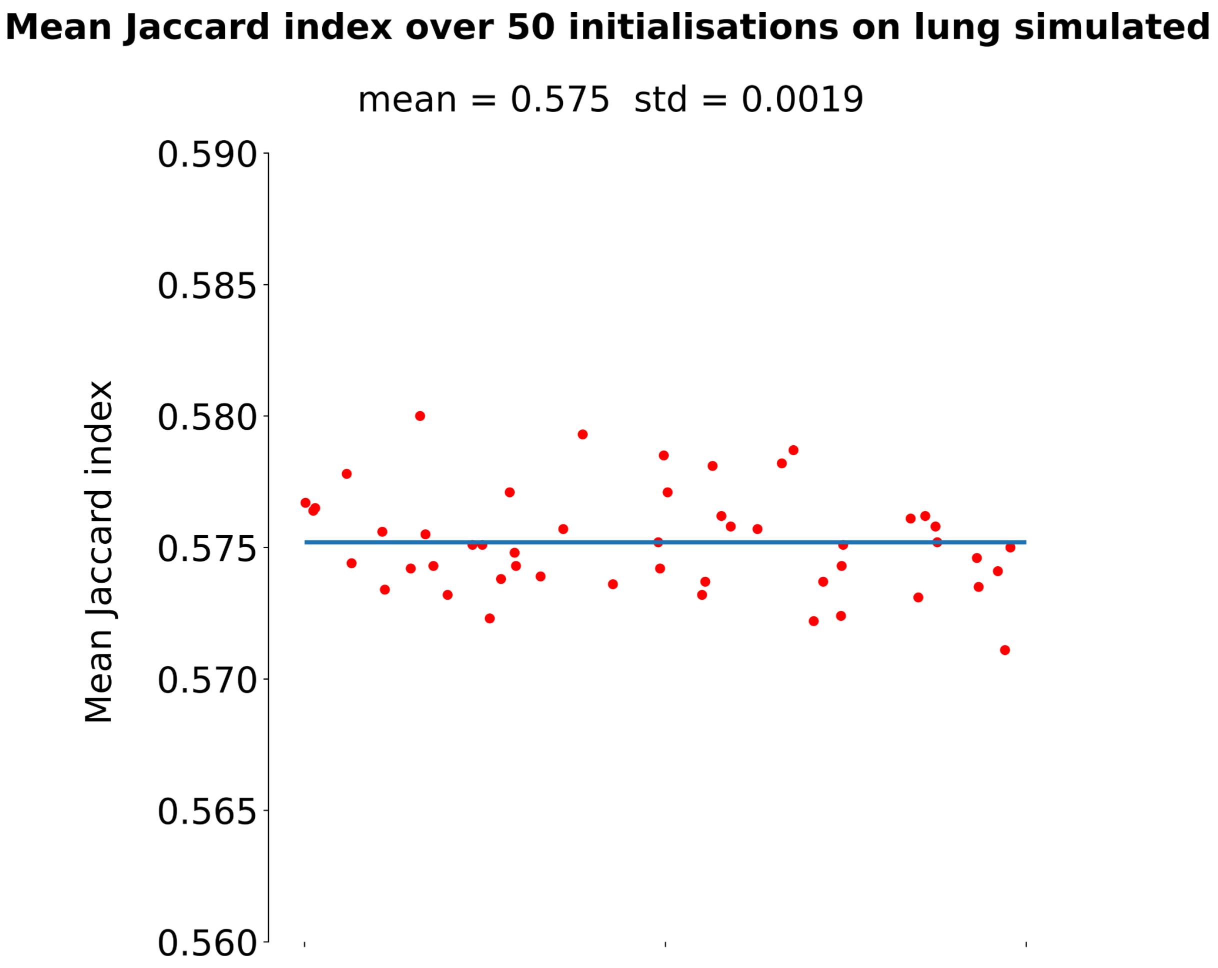

Supplementary Figure 6: The mean Jaccard index per cell obtained for 50 different random initializations of ComSeg on our simulated dataset of lung tissue. The blue line is the mean of all the Jaccard index obtained for 50 different random initializations.

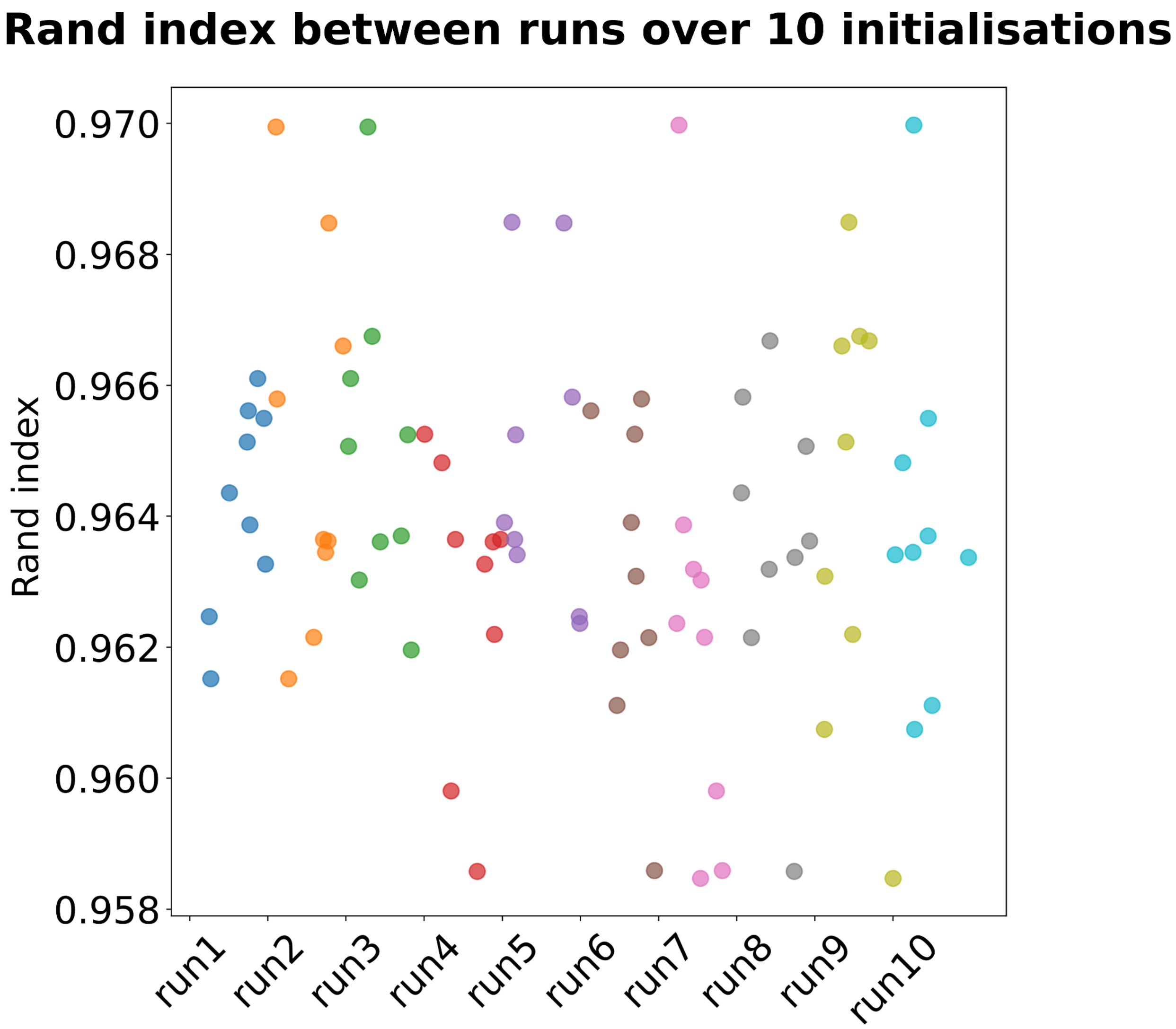

Supplementary Figure 7: Each spot corresponds to the rand index between two different initializations of ComSeg on the simulated dataset. The color of the spots correspond to the run taken as reference. The rand index can be interpreted here as the proportion of RNA in the same cell for the pair compared runs.

Supplementary Tables

Supplementary table 1 : Runtime and memory usage analysis for different methods on the mouse ileum dataset. Experiments were done on a laptop with a 16 cores CPU Intel(R) Core(TM) i9-10885H CPU @ 2.40GHz and with a GPU Quadro T2000 with 4Go of memory. The memory requirement was measured with the *memory_profiler* pip package.

	ComSeg	Watershed	pciSeq	Baysor	SCS*
Memory requirement	1,34Go	0,52Go	2,83Go	8,38Go	6.32go
Time	29 minutes	1 minutes	3 minutes	29 minutes	51 minutes
GPU	No	No	No	No	Yes

*for SCS, we divided SCS into crops of 3000x3000 pixels so the training fits our laptop memory and GPU.

References

1. Petukhov, V. *et al.* Cell segmentation in imaging-based spatial transcriptomics. *Nat. Biotechnol.* 40, 345–354 (2022).

2. Stringer, C., Wang, T., Michaelos, M. & Pachitariu, M. Cellpose: a generalist algorithm for cellular segmentation. *Nat. Methods* **18**, 100–106 (2021).

3. Turgay, Y. *et al.* The molecular architecture of lamins in somatic cells. *Nature* 543, 261–264 (2017).

4. Curras-Alonso, S. *et al.* An interactive murine single-cell atlas of the lung responses to radiation injury. *Nat. Commun.* **14**, 2445 (2023).
