# Supplementary material for: A point cloud segmentation framework for image-based spatial transcriptomics
Source: Commun Biol. 2024 Jul 6;7:823. doi: 10.1038/s42003-024-06480-3 (PMC11227573; doi:10.1038/s42003-024-06480-3)
Supplement: Supplementary file 1 — Peer Review File [file 42003_2024_6480_MOESM1_ESM.pdf]

nature portfolio

Peer Review File

A point cloud segmentation framework for image-based spatial transcriptomics

Open Access This file is licensed under a Creative Commons Attribution 4.0 International License, which permits use, sharing, adaptation, distribution and reproduction in any medium or format, as long as you give appropriate credit to the original author(s) and the source, provide a link to the Creative Commons license, and indicate if changes were made. In the cases where the authors are anonymous, such as is the case for the reports of anonymous peer reviewers, author attribution should be to 'Anonymous Referee' followed by a clear attribution to the source work. The images or other third party material in this file are included in the article's Creative Commons license, unless indicated otherwise in a credit line to the material. If material is not included in the article's Creative Commons license and your intended use is not permitted by statutory regulation or exceeds the permitted use, you will need to obtain permission directly from the copyright holder. To view a copy of this license, visit http://creativecommons.org/licenses/by/4.0/.

Reviewers' comments:

Reviewer #1 (Remarks to the Author):

he authors introduce ComSeg, a segmentation algorithm that directly operates on single RNA positions without relying on predefined assumptions about cell shape or external datasets. While the paper is well-organized, there is a need for simplifying the notation in the method section, particularly in the Spatial Correlation Computation section, to enhance readability due to an excessive use of symbols and functions. Additionally, the method draws inspiration from downstream analysis algorithms, and it is recommended that the authors cite and discuss key ideas, such as the utilization of KNN graphs and transcriptomic similarity, as well as the variation in the weight assigned to space (doi.org/10.1038/s41467-023-36796-3, doi.org/10.1093/gigascience/giac075, doi.org/10.1093/gigascience/giac064, 10.1038/s41592-021-01358-2).
Comprehensive evaluations conducted on both simulated and real datasets illustrate that ComSeg outperforms existing methods in in-situ single-cell RNA profiling and cell type identification. To strengthen the experiments, it is suggested to include more real experiments and explore the tuning of the k parameter across diverse datasets.

Reviewer #2 (Remarks to the Author):

This manuscript proposes a segmentation framework (ComSeg) for image-based spatial transcriptomes. It operates directly on single RNA positions without implicit or explicit priors on cell shape. This work focuses on a key problem in current spatial transcriptome research, that is, how to assign molecules to right cells. However, this manuscript has limitations in the performance validation experiments of ComSeg. Some important experiments and comparisons need to be further considered. The detailed comments are listed as follows.

1. In the "Introduction" section, the review of existing segmentation methods is not complete. Although the overall shortcomings of existing methods are explained, there is no explanation or authors' perspective on the details, such as advantages and disadvantages, of each method. Some state-of-the-art segmentation methods are not mentioned, such as SCS (Chen H. et al. (2023), doi:10.1038/s41592-023-01939-3).

2. Most of the experiments are performed on the simulation datasets, which is generated based on "SimTissue". And is "SimTissue" a proven and publicly recognized tool? If not, how to ensure the reliability of segmentation experiment results?

3. The experimental dataset is relatively small, containing data from only two lung tissues. This has

certain limitations. Considering that there may be differences in molecular density and cell morphology in different tissues, in order to more comprehensively evaluate the performance of different methods, experiments on more different tissue datasets derived from different platforms are needed.

4. In addition to Watershed, pciSeq and Baysor, the authors should consider comparative experiments with more state-of-the-art methods, such as SCS.

5. The application scope of ComSeg appears to be limited to image-based spatial transcriptomics. However, sequencing-based spatial transcriptomics data also provides molecular positional information, and some methods like stereo-seq even offer additional information such as nuclear masks. Can ComSeg be applied to sequencing-based spatial transcriptomics data as well? If not, what are the reasons for its inapplicability to such data? And, can ComSeg be adapted or modified to handle sequencing-based spatial transcriptomics data?

6. The experiments lack clear explanation and comparison regarding the time and resource consumption of different methods. In practice, some methods may face challenges when dealing with datasets of larger tissue areas and higher resolutions, leading to problems such as out of memory, abnormal interruptions and prolonged wait times for results. These considerations are of utmost importance for users, and the authors should provide comparisons and explanations on resource configuration and runtime.

7. Other minor issues.
(1) It is recommended that the content in the first column of Table 1 be formatted uniformly, that is, the first letter of “convex” should be capitalized.
(2) It is recommended that the titles of the first subsections of “Results” and “Material and Methods” sections be distinguished.

Reviewer #3 (Remarks to the Author):

The field of spatially resolved transcriptomics is rapidly expanding. Despite the emergence of several new biological protocols, there has been a lack of development in novel methods for data analysis. Accurate cell segmentation is an area that has been neglected and most of the existing methods aim to improve the existing staining-based segmentation. As a result, they are unable to perform the segmentation de-novo. However, the authors have proposed a new method called CompSeg, which requires minimal assumptions about data and relies solely on molecule density and gene composition. It uses a novel use of Leiden clustering for spatial data segmentation using local gene expression patterns, which were introduced in [1] and [4]. The method is implemented in Python and is well-documented. Unfortunately, the authors do not provide support for the claim that the method does not require additional data (see Major comment #5).

To validate the package, the authors created a simulation framework called SimTissue, and utilized it to compare the segmentation methods on various toy scenarios. The majority of the paper focuses on these simulation benchmarks (see Major comment #1) with the rest of the results shown on two in-house datasets (see Major comment #3). Although the quality of these benchmarks is questionable (see the comments), it's always challenging to benchmark your own methods.

I recommend accepting the manuscript after the major comments are fixed, aiming that independent researchers would later do more extensive benchmarking of various segmentation methods.

Major comments

1. The major part of the paper is devoted to comparing the existing segmentation methods on various simulations. On one side these results, as well as the simulation package, have theoretical value for segmentation method developers. They show important corner cases and help to test the segmentation methods on those. On the other, the applicability of these results to real-world scenarios is unclear. For example, "Sim 1&2" includes only two genes, "Sim 1-4" have square cell shapes and Sim 5 has L-shapes. The fact that the Watershed algorithm shows the best results on these benchmarks and nearly the worst results on the real-world data is also indicating that these benchmarks don't generalize to real problems. Benchmarking the method on more popular scenarios (such as MERFISH, 10x Xenium, or Nanostring CosmX) would be much more valuable. So, the benchmarking should focus on real-world data. I would even suggest moving most of the simulation results to a Supplementary Note.
2. The CompSeg method relies heavily on the assumption of homogeneous expression in a cell, which was shown to often be wrong [1], [2]. The performance of the package must be demonstrated on such a dataset. For example, the Mouse Ileum dataset from [1] has a particularly complex sub-cellular structure of Enterocytes, showing both a strong nuclei signal and expression polarization. Many other modern publically available datasets, such as those from Nanostring CosmX (https://nanostring.com/products/cosmx-spatial-molecular-imager/ffpe-dataset/) and VizGen (https://vizgen.com/data-release-program/) have pronounced nuclei signal.
3. The datasets used for the performance demonstration have an uncharacteristically small number of measured genes (34 and 147). Modern protocols, such as 10x Xenium and Nanostring CosmX measure ~1000 genes, with the most recent data having ~6000 genes. Showing the performance with those settings would be much more informative for potential users.
4. Additionally, most of the modern protocols (including Xenium, VizGen, and CosmX) measure the membrane signal. While it is far from perfect and doesn't always cover all the slices, it should be possible to find a few fields of view with a high-quality membrane signal and use them for benchmarking. In some scenarios, it can and should be used as ground truth.
5. The authors claim in multiple places (including Table 1, page 3 par. 2, page 16 par.2) that ComSeg doesn't require additional data, such as DAPI stains. However, in practice, the method does require cell centroids, which is stated in Methods, page 20 ("While cell or nuclei landmarks are optional, these cell centroid nodes are required to estimate the single-cell RNA profiles"). As the authors

explain it on page 19: "the landmarks can also originate from other stainings [...] or the analysis of RNA density or any other method". The problem, however, is that the analysis of RNA density was only used for identifying cell centroids in early works [3], and hasn't been used since then because of its poor performance. To my knowledge, staining segmentation is the only actively used method to identify cell centroids. Which makes the claim that "CompSeg doesn't require stainings" misleading. I would strongly recommend either withdrawing this claim or introducing a method for cell centroid detection, showing its performance and including it as a part of the package.
6. On all the benchmarks, the authors optimize the parameters of their method for each dataset separately, while keeping the parameters of other methods to defaults (see Supplementary Methods). It is not a fair comparison, and this should at least be noted in the main article.

Points 1-4 can all be covered with a demonstration on one or two datasets with a high number of genes, membrane signal, and sub-cellular structure. However, I would suggest also showing the sub-cellular structure part on the Mouse Ileum dataset, as it's a particularly tricky one.

Minor comments

1. The authors claim (page 6) that "Baysor and pciSeq both match their cell shape prior to the RNA spatial distribution to maximize a likelihood function. However, this strategy may fail on sparse data." However, both methods were tested on sparse HybISS data and showed no problems. And, in the case of pciSeq, it was specifically developed for ISS data. Support for this claim would be appreciated.
2. In Supplementary Note 1, the authors evaluate the robustness of their algorithm by showing that regardless of the random seed it achieves nearly the same performance on their benchmarks. A more natural metric would be to show whether the algorithm converges to the same set of labels per cell. For example, by measuring the Adjusted Rand Index between their runs. An overview of other alternatives for label assignment comparison can be found in the scikit-learn documentation: https://scikit-learn.org/stable/modules/clustering.html#clustering-performance-evaluation.
3. The authors propose "proximity weighted expression vectors", which use molecule neighborhoods for segmentation-free evaluation of expression patterns for building a weighted k-NN graph. This idea was previously proposed in [1] under the name of Neighborhood Composition Vectors. So, adding a citation to that would be appropriate.
4. The authors don't provide the code to reproduce their benchmarks. Publishing it would help with the reproducibility of their work.
5. In all the examples of segmentation by different methods (e.g., Fig. 1a, Fig. 4a) the authors omit molecules, not assigned to any cell. It makes it harder to interpret the performance of an individual method. Showing non-assigned molecules with a separate color or shape would improve interpretability.
6. The authors don't show any biological results of their segmented data. It would be nice to have a supplement figure with some UMAP plots showing cell embeddings as a sanity check.

References

1. Petukhov, V., Xu, R.J., Soldatov, R.A. et al. Cell segmentation in imaging-based spatial

transcriptomics. Nat Biotechnol 40, 345–354 (2022). https://doi.org/10.1038/s41587-021-01044-w
2. Bento: A toolkit for subcellular analysis of spatial transcriptomics data. Clarence K. Mah, Noorsher Ahmed, Nicole Lopez, Dylan Lam, Alexander Monell, Colin Kern, Yuanyuan Han, Gino Prasad, Anthony J. Cesnik, Emma Lundberg, Quan Zhu, Hannah Carter, Gene W. Yeo. bioRxiv 2022.06.10.495510; doi: https://doi.org/10.1101/2022.06.10.495510
3. Codeluppi, S., Borm, L.E., Zeisel, A. et al. Spatial organization of the somatosensory cortex revealed by osmFISH. Nat Methods 15, 932–935 (2018). https://doi.org/10.1038/s41592-018-0175-z
4. Park, J., Choi, W., Tiesmeyer, S. et al. Cell segmentation-free inference of cell types from in situ transcriptomics data. Nat Commun 12, 3545 (2021). https://doi.org/10.1038/s41467-021-23807-4

General reply

We thank the reviewers for their comments. It is our pleasure to submit an extensively revised manuscript, which addresses all raised concerns and questions.

Below we provide detailed answers to all comments in blue italic text.

REVIEWERs COMMENT ARE ORDERED AS 3 , 2, 1

Reviewer #3

The field of spatially resolved transcriptomics is rapidly expanding. Despite the emergence of several new biological protocols, there has been a lack of development in novel methods for data analysis. Accurate cell segmentation is an area that has been neglected and most of the existing methods aim to improve the existing staining-based segmentation. As a result, they are unable to perform the segmentation de-novo. However, the authors have proposed a new method called ComSeg, which requires minimal assumptions about data and relies solely on molecule density and gene composition. It uses a novel use of Leiden clustering for spatial data segmentation using local gene expression patterns, which were introduced in [1] and [4]. The method is implemented in Python and is well-documented. Unfortunately, the authors do not provide support for the claim that the method does not require additional data (see Major comment #5).

To validate the package, the authors created a simulation framework called SimTissue, and utilized it to compare the segmentation methods on various toy scenarios. The majority of the paper focuses on these simulation benchmarks (see Major comment #1) with the rest of the results shown on two in-house datasets (see Major comment #3). Although the quality of these benchmarks is questionable (see the comments), it's always challenging to benchmark your own methods.

I recommend accepting the manuscript after the major comments are fixed, aiming that independent researchers would later do more extensive benchmarking of various segmentation methods.

Major comments

R3.1. The major part of the paper is devoted to comparing the existing segmentation methods on various simulations. On one side these results, as well as the simulation package, have theoretical value for segmentation method developers. They show important corner cases and

help to test the segmentation methods on those. On the other, the applicability of these results to real-world scenarios is unclear. For example, "Sim 1&2" includes only two genes, "Sim 1-4" have square cell shapes and Sim 5 has L-shapes. The fact that the Watershed algorithm shows the best results on these benchmarks and nearly the worst results on the real-world data is also indicating that these benchmarks don't generalize to real problems. Benchmarking the method on more popular scenarios (such as MERFISH, 10x Xenium, or Nanostring CosmX) would be much more valuable. So, the benchmarking should focus on real-world data. I would even suggest moving most of the simulation results to a Supplementary Note.

We thank the reviewer for this comment. It is true that some of the simulations were not meant to generalize directly to real-world datasets, but rather aimed at investigating underlying hypotheses and failure modes of the benchmarked methods. For instance, the L-shape simulation with a few genes demonstrates that even in a very simple case, certain methods lead to suboptimal results. However, we agree that a benchmark on real experimental data would make the paper much stronger. Based on the reviewers comments, we have now added substantial new results on more diverse experimental data (see comments **R.3.2, R3.3, R3.4** below), and moved our simulation results as suggested to the supplement.

R3.2. The ComSeg method relies heavily on the assumption of homogeneous expression in a cell, which was shown to often be wrong [1], [2]. The performance of the package must be demonstrated on such a dataset. For example, the Mouse Ileum dataset from [1] has a particularly complex sub-cellular structure of Enterocytes, showing both a strong nuclei signal and expression polarization. Many other modern publically available datasets, such as those from Nanostring CosmX (https://nanostring.com/products/cosmx-spatial-molecular-imager/ffpe-dataset/) and VizGen (https://vizgen.com/data-release-program/) have pronounced nuclei signal.

As suggested, we applied ComSeg to the Mouse ileum dataset. For more comprehensive validation, we also established a ground-truth on these data based on areas with high-quality staining of the cellular membrane (see **R3.4** for more details). This analysis revealed that ComSeg yields high-quality results compared to the other benchmarked methods (see new Figure 4, **page 12 lines 346-356**). This shows that ComSeg can also be applied to cells with preferential subcellular location, as present in the Mouse ileum dataset and shown in the new Supplementary Figure 4. We agree however, that some subcellular localization patterns would negatively impact the algorithm's performance. On the other hand, this is true for all methods that operate on RNA locations, as the underlying hypothesis is that significant changes in expression profiles indicate the presence of a cell boundary. In addition to conducting this analysis, we now also provide the annotated ground truth data, which we expect to be of high value to the scientific community for future benchmarking studies. Our annotated ground truth data publicly available at https://zenodo.org/records/11067800

R3.3. The datasets used for the performance demonstration have an uncharacteristically small number of measured genes (34 and 147). Modern protocols, such as 10x Xenium and Nanostring CosmX measure ~1000 genes, with the most recent data having ~6000 genes. Showing the performance with those settings would be much more informative for potential users.

In addition to the above mentioned Mouse ileum dataset, we added a Vizgen MERFISH human breast cancer dataset to our benchmark. This dataset has 550 different RNAs imaged on a 10mm X 8mm tissue section. For the purpose of this study, we analyzed a 2D subsection of 1900 × 970 µm. We also created ground truth from areas with high quality staining (see **R3.4** for more details). Again, ComSeg yielded excellent results, showing its capability to effectively analyze datasets with a high number of genes. Our annotated Ground truth is also publicly available at https://zenodo.org/records/11067800.

R3.4. Additionally, most of the modern protocols (including Xenium, VizGen, and CosmX) measure the membrane signal. While it is far from perfect and doesn't always cover all the slices, it should be possible to find a few fields of view with a high-quality membrane signal and use them for benchmarking. In some scenarios, it can and should be used as ground truth.

We thank the reviewer for this suggestion. The Mouse Ileum dataset and the MERFISH human breast cancer dataset indeed display areas with high quality cell membrane staining. We implemented an analytical workflow to automatically create ground truth data from these areas (see **page 11 lines 332-344** and Material & Methods, **page 23 lines 768-783** for more detail). First, we use the deep-learning based approach Cellpose to segment both the nuclei and the cell membrane. Second, we automatically select areas with high quality membrane staining, by only using cells that have one nucleus within their segmented boundary. With this approach, we obtained a ground truth consisting of 668 cells for the Ileum data-set and 2061 annotated cells for the Vizgen breast cancer dataset. These data are also publicly available at https://zenodo.org/records/11067800.
Besides, results of our benchmarking experiments on high quality data with a membrane staining confirm the good performance of ComSeg. Indeed, ComSeg performs equally well or better as compared to the competing methods on this additional benchmark in terms of Jaccard index. The segmentation results of all the methods of our benchmark of these two datasets is also publicly available at https://zenodo.org/records/11067800.

R3.5. The authors claim in multiple places (including Table 1, page 3 par. 2, page 16 par.2) that ComSeg doesn't require additional data, such as DAPI stains. However, in practice, the method does require cell centroids, which is stated in Methods, page 20 ("While cell or nuclei landmarks are optional, these cell centroid nodes are required to estimate the single-cell RNA profiles"). As the authors explain it on page 19: "the landmarks can also originate from other stainings [...] or the analysis of RNA density or any other method". The problem, however, is that the analysis of RNA density was only used for identifying cell centroids in early works [3], and hasn't been used

since then because of its poor performance. To my knowledge, staining segmentation is the only actively used method to identify cell centroids. Which makes the claim that "ComSeg doesn't require stainings" misleading. I would strongly recommend either withdrawing this claim or introducing a method for cell centroid detection, showing its performance and including it as a part of the package.

We agree with the reviewer: this might indeed be misleading. While ComSeg could in principle be used without additional data and only rely on the transcriptional profiles, we never used this possibility in practice. We therefore followed the reviewer's advice and removed the claim that ComSeg doesn't require stainings (**Page 3, line 111; page 14 line 373, pages 18 line 559, pages 19 lines 609** of the revised manuscript) and added this requirement in table one.

R3.6.. On all the benchmarks, the authors optimize the parameters of their method for each dataset separately, while keeping the parameters of other methods to defaults (see Supplementary Methods). It is not a fair comparison, and this should at least be noted in the main article.

We agree with the reviewer that fair comparisons are always a challenge. This is particularly true for new data modalities or new analysis tasks. The fairest way of comparing methods is actually the organization of a challenge, such that all participants have the possibility to tune their methods in the best possible way.

However, we believe that our comparison is reasonably fair. Indeed, there are few hyperparameters that need to be set, most of which are related to the size of the nucleus or the expected size of the cell. In particular, there was no hyperparameter tuning of ComSeg other than two distance parameters that directly relate to the expected cell size and geometry. This kind of optimization was also performed on competing methods, such as Baysor, where the scaling parameter is updated (albeit automatically) for each new dataset. Moreover, as the Mouse Ileum dataset was published together with Baysor, we used the precise parameter setting from the original article, and can therefore assume that the parameters were optimally set in this case.

We added a more detailed description of the parameters used on page 6 (lines 183-196) and in Supplementary Note 1. Additionally, we highlight the current challenge of benchmarking methods in the discussion (lines 402-405) : "As the number of methods and datasets continues to increase, finding optimal parameters for each of them to obtain fair comparison between them becomes increasingly challenging. We believe that independent benchmark studies for spatial RNA profiling data would be beneficial for the community."

R3.7. Points 1-4 can all be covered with a demonstration on one or two datasets with a high number of genes, membrane signal, and sub-cellular structure. However, I would suggest also showing the sub-cellular structure part on the Mouse Ileum dataset, as it's a particularly tricky one.

We thank the reviewer for the constructive criticism and helpful suggestion. We applied ComSeg to the Mouse Ileum dataset (Fig 4) containing RNA with intra-cellular preferential distribution (Supplementary Figure 4). ComSeg demonstrates strong performance on this dataset (Fig. 4c). In addition, our experiments demonstrate that comSeg is able to assign genes to the right cell even for genes with intra-cellular preferential distribution (Supplementary Figure 4).

Minor comments

R3.8 The authors claim (page 6) that "Baysor and pciSeq both match their cell shape prior to the RNA spatial distribution to maximize a likelihood function. However, this strategy may fail on sparse data." However, both methods were tested on sparse HybISS data and showed no problems. And, in the case of pciSeq, it was specifically developed for ISS data. Support for this claim would be appreciated.

We agree with the reviewer and removed this claim (**page 6**).

R3.9. In Supplementary Note 1, the authors evaluate the robustness of their algorithm by showing that regardless of the random seed it achieves nearly the same performance on their benchmarks. A more natural metric would be to show whether the algorithm converges to the same set of labels per cell. For example, by measuring the Adjusted Rand Index between their runs. An overview of other alternatives for label assignment comparison can be found in the scikit-learn documentation: https://scikit-learn.org/stable/modules/clustering.html#clustering-performance-evaluation.

We thank the reviewer for this suggestion, and we added this evaluation to the manuscript (Supplementary Note 3, new Supplementary Figure 7). In short, we computed the Rand index on the RNA cell assignment between 10 different initialisations of ComSeg. This analysis shows that the Rand index between pairs of runs is stable with a value of approx 0.96, indicating that 96% of the RNA are grouped into a similar way, i.e either in the same cell or either in different cell, across different initializations.

R3.10. The authors propose "proximity weighted expression vectors", which use molecule neighborhoods for segmentation-free evaluation of expression patterns for building a weighted k-NN graph. This idea was previously proposed in [1] under the name of Neighborhood Composition Vectors. So, adding a citation to that would be appropriate.

We now include a reference to the Neighborhood Composition Vectors on **page 16, lines 487-489**. Neighborhood Composition Vectors are akin to our suggested "proximity weighted expression vectors," with the difference that our proposition incorporates a distance weight.

R3.11 The authors don't provide the code to reproduce their benchmarks. Publishing it would help with the reproducibility of their work.

We apologize for this oversight. While the code was available (https://zenodo.org/records/10222671), we failed to properly reference this in the manuscript. We have added the reference. In addition, we now provide both complete code to reproduce the analyses (https://github.com/tdefa/script_Benchmark) as well as the newly generated ground-truth data and the corresponding segmentation result of each all benchmarked methods (https://zenodo.org/records/11067800)

R3.12. In all the examples of segmentation by different methods (e.g., Fig. 1a, Fig. 4a) the authors omit molecules, not assigned to any cell. It makes it harder to interpret the performance of an individual method. Showing non-assigned molecules with a separate color or shape would improve interpretability.

We thank the reviewer for his suggestion. We have now added cross to represent non-assigned RNA on Fig. 2, Fig. 3 and Fig. 4.

R3.13. The authors don't show any biological results of their segmented data. It would be nice to have a supplement figure with some UMAP plots showing cell embeddings as a sanity check.

As suggested by the reviewer, we plotted such Umap clustering of the estimated single cell profile from each method for the mouse ileum dataset :

Umap from the segmentation of the mouse ileum dataset for the different methods

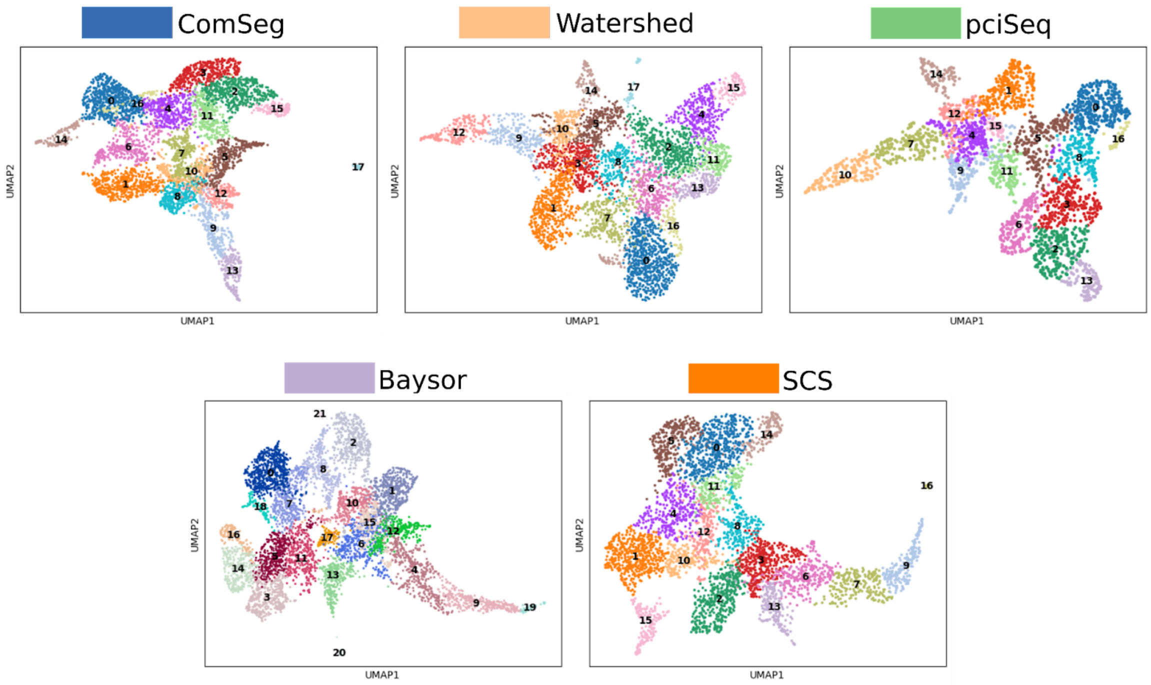

Figure : Clustering with leiden and Umap visualization of the single cell profile obtained for each benchmarked method on the mouse ileum dataset.

References

1. Petukhov, V., Xu, R.J., Soldatov, R.A. et al. Cell segmentation in imaging-based spatial transcriptomics. Nat Biotechnol 40, 345–354 (2022). https://doi.org/10.1038/s41587-021-01044-w
2. Bento: A toolkit for subcellular analysis of spatial transcriptomics data. Clarence K. Mah, Noorsher Ahmed, Nicole Lopez, Dylan Lam, Alexander Monell, Colin Kern, Yuanyuan Han, Gino Prasad, Anthony J. Cesnik, Emma Lundberg, Quan Zhu, Hannah Carter, Gene W. Yeo. bioRxiv 2022.06.10.495510; doi: https://doi.org/10.1101/2022.06.10.495510
3. Codeluppi, S., Borm, L.E., Zeisel, A. et al. Spatial organization of the somatosensory cortex revealed by osmFISH. Nat Methods 15, 932–935 (2018). https://doi.org/10.1038/s41592-018-0175-z
4. Park, J., Choi, W., Tiesmeyer, S. et al. Cell segmentation-free inference of cell types from in situ transcriptomics data. Nat Commun 12, 3545 (2021). https://doi.org/10.1038/s41467-021-23807-4

Reviewer #2

This manuscript proposes a segmentation framework (ComSeg) for image-based spatial transcriptomes. It operates directly on single RNA positions without implicit or explicit priors on cell shape. This work focuses on a key problem in current spatial transcriptome research, that is, how to assign molecules to right cells. However, this manuscript has limitations in the performance validation experiments of ComSeg. Some important experiments and comparisons need to be further considered. The detailed comments are listed as follows.

R2.1. In the "Introduction" section, the review of existing segmentation methods is not complete. Although the overall shortcomings of existing methods are explained, there is no explanation or authors' perspective on the details, such as advantages and disadvantages, of each method. Some state-of-the-art segmentation methods are not mentioned, such as SCS (Chen H. et al. (2023), doi:10.1038/s41592-023-01939-3).

We completed the description of the existing methods (**page 2, lines 85-90**) and the Table 1 adding reference to the latest published methods as suggested by the reviewer.

R2.2.. Most of the experiments are performed on the simulation datasets, which is generated based on "SimTissue". And is "SimTissue" a proven and publicly recognized tool? If not, how to ensure the reliability of segmentation experiment results?

SimTissue is a simulation framework we specifically developed for this study. As also discussed in R3.1, the goal of these simulations is to provide a well-controlled environment to test different scenarios with increasing complexity. This can thus provide an illustration of relatively simple corner cases and allow to study failure modes of the benchmarked methods.
Based on the suggestion of reviewer 1, we now moved these simulations to the Supplement and added two experimental data-sets (**R3.2** and **R3.3**). Importantly, we also created ground-truth data for these experimental datasets, permitting extensive benchmarking on real data. We believe that this ground truth data might be an additional useful result of our work and we therefore made it publicly available at https://zenodo.org/records/10172316.

R2.3.. The experimental dataset is relatively small, containing data from only two lung tissues. This has certain limitations. Considering that there may be differences in molecular density and cell morphology in different tissues, in order to more comprehensively evaluate the performance of different methods, experiments on more different tissue datasets derived from different platforms are needed.

As also suggested by reviewer 3 (**R3.2, R3.3, R3.4**), we extended our benchmark to two additional experimental data-sets (MERFISH Mouse Ileum, VizGen breast cancer). We provide a new Fig 4 and Supplementary Figure 4.

R2.4.. In addition to Watershed, pciSeq and Baysor, the authors should consider comparative experiments with more state-of-the-art methods, such as SCS.

As suggested, we added SCS to our benchmark. SCS has been developed and applied for data-sets with large transcriptome coverage. It has been shown to outperform state-of-the-art methods in the analysis of sequencing-based data (Stereo-Seq) and a MerFISH data-set with 4000 targeted genes. However, we could not confirm this in our benchmarking: we observed that SCS performs worse than the other methods tested. This might be due to insufficient training data, or due to the fact that SCS has been designed for IST methods with large coverage. While such coverage can be achieved by latest generation approaches, many datasets (as the ones also benchmarked here) display less than 1000 genes). The relatively low scores obtained for SCS in our study are thus potentially due to the lower number of genes that are present in the used data. So SCS seems therefore better suited to analyze data with several thousands targeted genes whereas ComSeg is also adapted to small scale study with few genes as demonstrated in our benchmark. Another explanation could be that SCS does not utilize 3D information and that spatial resolution had to be reduced to accommodate SCS to image based data.

R2.5.. The application scope of ComSeg appears to be limited to image-based spatial transcriptomics. However, sequencing-based spatial transcriptomics data also provides molecular positional information, and some methods like stereo-seq even offer additional information such as nuclear masks. Can ComSeg be applied to sequencing-based spatial transcriptomics data as well? If not, what are the reasons for its inapplicability to such data? And, can ComSeg be adapted or modified to handle sequencing-based spatial transcriptomics data?

ComSeg operates on point clouds and relies thus on the positions of single RNA molecules, which are not available in sequencing based spatial transcriptomics. For this reason, ComSeg cannot be directly applied to sequencing-based spatial transcriptomics (SST) data. We also do not see a straightforward extension to make ComSeg applicable to SST data.
We added this limitation to the discussion (**page 16, lines 403-405**). For sequencing based data, approaches specifically designed for this modality, such as the above mentioned SCS, are preferable.

R2.6.. The experiments lack clear explanation and comparison regarding the time and resource consumption of different methods. In practice, some methods may face challenges when dealing with datasets of larger tissue areas and higher resolutions, leading to problems such as out of memory, abnormal interruptions and prolonged wait times for results. These

considerations are of utmost importance for users, and the authors should provide comparisons and explanations on resource configuration and runtime.

We thank the reviewer for this suggestion. We now display the run time and memory requirement of each method on the mouse ileum dataset using a standard laptop (new **Supplementary table 1**). In particular, ComSeg required 1.34 Go of memory and the analysis took 29 minutes. ComSeg is slower than pciSeq and watershed but have the same run time than Baysor and is faster than SCS.

R2.7.. Other minor issues.
(1) It is recommended that the content in the first column of Table 1 be formatted uniformly, that is, the first letter of “convex” should be capitalized.

This is now corrected.

(2) It is recommended that the titles of the first subsections of “Results” and “Material and Methods” sections be distinguished.

We now changed the heading the Material & Methods to have distinct titles.

Reviewer #1

The authors introduce ComSeg, a segmentation algorithm that directly operates on single RNA positions without relying on predefined assumptions about cell shape or external datasets. While the paper is well-organized, there is a need for simplifying the notation in the method section, particularly in the Spatial Correlation Computation section, to enhance readability due to an excessive use of symbols and functions. Additionally, the method draws inspiration from downstream analysis algorithms, and it is recommended that the authors cite and discuss key ideas, such as the utilization of KNN graphs and transcriptomic similarity, as well as the variation in the weight assigned to space (doi.org/10.1038/s41467-023-36796-3, doi.org/10.1093/gigascience/giac075, doi.org/10.1093/gigascience/giac064, 10.1038/s41592-021-01358-2).
Comprehensive evaluations conducted on both simulated and real datasets illustrate that ComSeg outperforms existing methods in in-situ single-cell RNA profiling and cell type identification. To strengthen the experiments, it is suggested to include more real experiments and explore the tuning of the k parameter across diverse datasets.

R1.1.“there is a need for simplifying the notation in the method section, particularly in the Spatial Correlation Computation section, to enhance readability due to an excessive use of symbols and functions.”

We thank the reviewer for this comment. We wanted to explain our approach in detail in the Methods section, such that every step is clear and formally correct. We believe that this is best done by putting the explicit formulae. However, we understand that our first version might not have been entirely clear, and we have therefore rewritten the paragraph, and we hope that it is clearer now.

R1.2 "Additionally, the method draws inspiration from downstream analysis algorithms, and it is recommended that the authors cite and discuss key ideas, such as the utilization of KNN graphs and transcriptomic similarity, as well as the variation in the weight assigned to space (doi.org/10.1038/s41467-023-36796-3, doi.org/10.1093/gigascience/giac075, doi.org/10.1093/gigascience/giac064, 10.1038/s41592-021-01358-2).

As suggested, we added a reference to existing work leveraging KNN to study spatial transcriptomic similarity **(Page 19, lines 596-600)**. We also discuss the clustering method of transcriptomic profile with weight assigned to space in the discussion, and outline how they could provide future improvement for ComSeg (**Page 15, lines 425-434**).

R1.3
To strengthen the experiments, it is suggested to include more real experiments

As suggested also by the other reviewers, we applied ComSeg to a MERFISH Mouse ileum dataset and a MERFISH breast cancer dataset (see **R3.2, R3.3, R3.4**). Results are shown in new Fig. 4 and new Supplementary figure 4.

R1.4 Explore the tuning of the k parameter across diverse datasets.

We now added a more detailed analysis of the effect of the parameter K (**Supplementary Figure 5**). ComSeg displayed stable performance with respect to variations in a reasonable range (from K=5 to K=20).

Reviewers' comments:

Reviewer #1 (Remarks to the Author):

The authors have addressed my comments.

Reviewer #2 (Remarks to the Author):

This manuscript has improved significantly over the previous version, with most comments being appropriately addressed. However, I still have one concern that needs further explanation from the authors. In R2.5, it is stated that 'ComSeg operates on point clouds and relies on the positions of single RNA molecules, which are not available in sequencing-based spatial transcriptomics.' I find this confusing as current sequencing-based spatial transcriptomics, such as stereo-seq and 10x Xenium, also provide highly precise data that includes the positions and expression levels of individual RNA molecules. Therefore, relying on the positions of single RNA molecules does not seem to limit ComSeg's application. I suggest the authors discuss this limitation and the potential reasons behind it in more detail in the "Discussion" section, to inform users who focus on data from various platforms. Additionally, on page 2, line 84, 'Method' should be 'Methods'. It is recommended that authors check the manuscript for writing issues.

Reviewer #3 (Remarks to the Author):

The authors fully addressed all the comments. So, I would recommend the manuscript to be published.

A minor issue is the lack of description of the simulation methods ("Methods" -> "Simulation Python package"). The current text describes high-level modeling scenarios, but not the statistical details of the models. Having those would make the package usage easier.

I'd also want to thank the authors for publishing the ground truth data for segmentation benchmarking. And if possible, it would be even better to deposit the trained CellPose models with those benchmarks.

General reply

We thank the reviewers for their comments. We are pleased to see that the reviewers were satisfied with the revised version of our article. We are happy to address the remaining issues below.

Of note, our latest version of ComSeg is now compatible with Sopa (https://gustaveroussy.github.io/sopa/) making the application of ComSeg on large datasets easier. Sopa is a processing pipeline for the analysis of image-based spatial-omics, built on top of SpatialData (https://spatialdata.scverse.org/). It is designed to be memory-efficient and scales to large datasets with millions of cells by managing the generation and aggregation of patches for cell segmentation.

Reviewers' comments:

Reviewer #1 (Remarks to the Author):

The authors have addressed my comments.

We thank the reviewer for the positive feedback.

Reviewer #2 (Remarks to the Author):

This manuscript has improved significantly over the previous version, with most comments being appropriately addressed. However, I still have one concern that needs further explanation from the authors. In R2.5, it is stated that 'ComSeg operates on point clouds and relies on the positions of single RNA molecules, which are not available in sequencing-based spatial transcriptomics.' I find this confusing as current sequencing-based spatial transcriptomics, such as stereo-seq and 10x Xenium, also provide highly precise data that includes the positions and expression levels of individual RNA molecules. Therefore, relying on the positions of single RNA molecules does not seem to limit ComSeg's application. I suggest the authors discuss this limitation and the potential reasons behind it in more detail in the “Discussion” section, to inform users who focus on data from various platforms. Additionally, on page 2, line 84, 'Method' should be 'Methods'. It is recommended that authors check the manuscript for writing issues.

We thank the reviewer for this comment and completed the discussion line 443-449 :
“Finally, ComSeg relies on the position of single RNA molecules which prevents its application on sequencing based spatial transcriptomic data with low spatial resolution. Indeed, the edge weights are defined with gene co-expression as similarity metrics. Applying ComSeg on sequencing based data with low spatial resolution would require defining an expression vector similarity metric between binned spots which might be an interesting extension. Finally, ComSeg

could in principle be used on sequencing based data if the spatial resolution is close to IST, such as Stereo-seq."

Reviewer #3 (Remarks to the Author):

The authors fully addressed all the comments. So, I would recommend the manuscript to be published.

A minor issue is the lack of description of the simulation methods ("Methods" -> "Simulation Python package"). The current text describes high-level modeling scenarios, but not the statistical details of the models. Having those would make the package usage easier.

I'd also want to thank the authors for publishing the ground truth data for segmentation benchmarking. And if possible, it would be even better to deposit the trained CellPose models with those benchmarks.

The RNA profiles are directly sampled from an external scRNA-seq dataset or fixed as constant. To make this point clearer, we replace "drawing" by "sampling" in the lines 682 and 683: "RNA expression levels can either be set as constant or be directly sampled from experimental measures, e.g. from scRNA-seq data. When sampling profiles from scRNA-seq, we sample for each simulated cell an expression profile of a single cell from scRNA-seq, then multiply the number of RNAs by a factor."
To further ease the use of our simulation package we released a new version of SimTissue with a complete documentation at https://simtissue.readthedocs.io.

We released a new version of our generated ground truth of the two MerFISH datasets and the corresponding benchmark segmentation results at https://zenodo.org/records/11237477. It now includes the used pretrained network and cellpose parameters for the MerFISH breast cancer dataset. For the mouse ileum dataset, we kept the fine-tuned Cellpose segmentation proposed in the original publication as indicated line 782.
